# Acute kidney injury in patients hospitalized with COVID-19 from the ISARIC WHO CCP-UK Study: a prospective, multicentre cohort study

**DOI:** 10.1093/ndt/gfab303

**Published:** 2021-10-18

**Authors:** Michael K Sullivan, Jennifer S Lees, Thomas M Drake, Annemarie B Docherty, Georgia Oates, Hayley E Hardwick, Clark D Russell, Laura Merson, Jake Dunning, Jonathan S Nguyen-Van-Tam, Peter Openshaw, Ewen M Harrison, J Kenneth Baillie, ISARIC4C Investigators, J Kenneth Baillie, Malcolm G Semple, Peter J M Openshaw, Gail Carson, Beatrice Alex, Benjamin Bach, Wendy S Barclay, Debby Bogaert, Meera Chand, Graham S Cooke, Annemarie B Docherty, Jake Dunning, Ana da Silva Filipe, Tom Fletcher, Christoper A Green, Ewen M Harrison, Julian A Hiscox, Antonia Ying Wai Ho, Peter W Horby, Samreen Ijaz, Saye Khoo, Paul Klenerman, Andrew Law, Wei Shen Lim, Alexander J Mentzer, Laura Merson, Alison M Meynert, Mahdad Noursadeghi, Shona C Moore, Massimo Palmarini, William A Paxton, Georgios Pollakis, Nicholas Price, Andrew Rambaut, David L Robertson, Clark D Russell, Vanessa Sancho-Shimizu, Janet T Scott, Thushan de Silva, Louise Sigfrid, Tom Solomon, Shiranee Sriskandan, David Stuart, Charlotte Summers, Richard S Tedder, Emma C Thomson, A A Roger Thompson, Ryan S Thwaites, Lance C W Turtle, Maria Zambon, Hayley Hardwick, Chloe Donohue, Ruth Lyons, Fiona Griffiths, Wilna Oosthuyzen, Lisa Norman, Riinu Pius, Thomas M Drake, Cameron J Fairfield, Stephen R Knight, Kenneth A Mclean, Derek Murphy, Catherine A Shaw, Jo Dalton, Michelle Girvan, Egle Saviciute, Stephanie Roberts, Janet Harrison, Laura Marsh, Marie Connor, Sophie Halpin, Clare Jackson, Carrol Gamble, Gary Leeming, Andrew Law, Murray Wham, Sara Clohisey, Ross Hendry, James Scott-Brown, William Greenhalf, Victoria Shaw, Sara McDonald, Seán Keating, Katie A Ahmed, Jane A Armstrong, Milton Ashworth, Innocent G Asiimwe, Siddharth Bakshi, Samantha L Barlow, Laura Booth, Benjamin Brennan, Katie Bullock, Benjamin W A Catterall, Jordan J Clark, Emily A Clarke, Sarah Cole, Louise Cooper, Helen Cox, Christopher Davis, Oslem Dincarslan, Chris Dunn, Philip Dyer, Angela Elliott, Anthony Evans, Lorna Finch, Lewis W S Fisher, Terry Foster, Isabel Garcia-Dorival, William Greenhalf, Philip Gunning, Catherine Hartley, Rebecca L Jensen, Christopher B Jones, Trevor R Jones, Shadia Khandaker, Katharine King, Robyn T Kiy, Chrysa Koukorava, Annette Lake, Suzannah Lant, Diane Latawiec, Lara Lavelle-Langham, Daniella Lefteri, Lauren Lett, Lucia A Livoti, Maria Mancini, Sarah McDonald, Laurence McEvoy, John McLauchlan, Soeren Metelmann, Nahida S Miah, Joanna Middleton, Joyce Mitchell, Shona C Moore, Ellen G Murphy, Rebekah Penrice-Randal, Jack Pilgrim, Tessa Prince, Will Reynolds, P Matthew Ridley, Debby Sales, Victoria E Shaw, Rebecca K Shears, Benjamin Small, Krishanthi S Subramaniam, Agnieska Szemiel, Aislynn Taggart, Jolanta Tanianis-Hughes, Jordan Thomas, Erwan Trochu, Libby van Tonder, Eve Wilcock, J Eunice Zhang, Lisa Flaherty, Nicole Maziere, Emily Cass, Alejandra Doce Carracedo, Nicola Carlucci, Anthony Holmes, Hannah Massey, Lee Murphy, Nicola Wrobel, Sarah McCafferty, Kirstie Morrice, Alan MacLean, Kayode Adeniji, Daniel Agranoff, Ken Agwuh, Dhiraj Ail, Erin L Aldera, Ana Alegria, Brian Angus, Abdul Ashish, Dougal Atkinson, Shahedal Bari, Gavin Barlow, Stella Barnass, Nicholas Barrett, Christopher Bassford, Sneha Basude, David Baxter, Michael Beadsworth, Jolanta Bernatoniene, John Berridge, Nicola Best, Pieter Bothma, David Chadwick, Robin Brittain-Long, Naomi Bulteel, Tom Burden, Andrew Burtenshaw, Vikki Caruth, David Chadwick, Duncan Chambler, Nigel Chee, Jenny Child, Srikanth Chukkambotla, Tom Clark, Paul Collini, Catherine Cosgrove, Jason Cupitt, Maria-Teresa Cutino-Moguel, Paul Dark, Chris Dawson, Samir Dervisevic, Phil Donnison, Sam Douthwaite, Ingrid DuRand, Ahilanadan Dushianthan, Tristan Dyer, Cariad Evans, Chi Eziefula, Chrisopher Fegan, Adam Finn, Duncan Fullerton, Sanjeev Garg, Sanjeev Garg, Atul Garg, Effrossyni Gkrania-Klotsas, Jo Godden, Arthur Goldsmith, Clive Graham, Elaine Hardy, Stuart Hartshorn, Daniel Harvey, Peter Havalda, Daniel B Hawcutt, Maria Hobrok, Luke Hodgson, Anil Hormis, Michael Jacobs, Susan Jain, Paul Jennings, Agilan Kaliappan, Vidya Kasipandian, Stephen Kegg, Michael Kelsey, Jason Kendall, Caroline Kerrison, Ian Kerslake, Oliver Koch, Gouri Koduri, George Koshy, Shondipon Laha, Steven Laird, Susan Larkin, Tamas Leiner, Patrick Lillie, James Limb, Vanessa Linnett, Jeff Little, Mark Lyttle, Michael MacMahon, Emily MacNaughton, Ravish Mankregod, Huw Masson, Elijah Matovu, Katherine McCullough, Ruth McEwen, Manjula Meda, Gary Mills, Jane Minton, Mariyam Mirfenderesky, Kavya Mohandas, Quen Mok, James Moon, Elinoor Moore, Patrick Morgan, Craig Morris, Katherine Mortimore, Samuel Moses, Mbiye Mpenge, Rohinton Mulla, Michael Murphy, Megan Nagel, Thapas Nagarajan, Mark Nelson, Matthew K O'Shea, Igor Otahal, Marlies Ostermann, Mark Pais, Selva Panchatsharam, Danai Papakonstantinou, Hassan Paraiso, Brij Patel, Natalie Pattison, Justin Pepperell, Mark Peters, Mandeep Phull, Stefania Pintus, Jagtur Singh Pooni, Frank Post, David Price, Rachel Prout, Nikolas Rae, Henrik Reschreiter, Tim Reynolds, Neil Richardson, Mark Roberts, Devender Roberts, Alistair Rose, Guy Rousseau, Brendan Ryan, Taranprit Saluja, Aarti Shah, Prad Shanmuga, Anil Sharma, Anna Shawcross, Jeremy Sizer, Manu Shankar-Hari, Richard Smith, Catherine Snelson, Nick Spittle, Nikki Staines, Tom Stambach, Richard Stewart, Pradeep Subudhi, Tamas Szakmany, Kate Tatham, Jo Thomas, Chris Thompson, Robert Thompson, Ascanio Tridente, Darell Tupper-Carey, Mary Twagira, Andrew Ustianowski, Nick Vallotton, Lisa Vincent-Smith, Shico Visuvanathan, Alan Vuylsteke, Sam Waddy, Rachel Wake, Andrew Walden, Ingeborg Welters, Tony Whitehouse, Paul Whittaker, Ashley Whittington, Padmasayee Papineni, Meme Wijesinghe, Martin Williams, Lawrence Wilson, Sarah Cole, Stephen Winchester, Martin Wiselka, Adam Wolverson, Daniel G Wooton, Andrew Workman, Bryan Yates, Peter Young, Malcolm G Semple, Antonia Ho, Patrick B Mark

**Affiliations:** Institute of Cardiovascular and Medical Sciences, University of Glasgow, Glasgow, UK; Institute of Cardiovascular and Medical Sciences, University of Glasgow, Glasgow, UK; Centre for Medical Informatics, Usher Institute, University of Edinburgh, Edinburgh, UK; Centre for Medical Informatics, Usher Institute, University of Edinburgh, Edinburgh, UK; Edinburgh Medical School, University of Edinburgh, Edinburgh, UK; HPRU in Infection and Emerging Diseases, Institute of Infection, Veterinary and Ecological Sciences, University of Liverpool, UK; Centre for Inflammation Research, University of Edinburgh, Edinburgh, UK; ISARIC Global Support Centre, University of Oxford, Oxford, UK; Centre for Tropical Medicine and Global Health, University of Oxford, Oxford, UK; Division of Epidemiology and Public Health, University of Nottingham, Nottingham, UK; National Heart and Lung Institute, Imperial College London, London, UK; Centre for Medical Informatics, Usher Institute, University of Edinburgh, Edinburgh, UK; Roslin Institute, University of Edinburgh, Edinburgh, UK; HPRU in Infection and Emerging Diseases, Institute of Infection, Veterinary and Ecological Sciences, University of Liverpool, UK; Medical Research Council-University of Glasgow Centre for Virus Research, Glasgow, UK; Institute of Cardiovascular and Medical Sciences, University of Glasgow, Glasgow, UK

**Keywords:** acute kidney injury, COVID-19, dialysis, renal failure, SARS-CoV-2

## Abstract

**Background:**

Acute kidney injury (AKI) is common in coronavirus disease 2019 (COVID-19). This study investigated adults hospitalized with COVID-19 and hypothesized that risk factors for AKI would include comorbidities and non-White race.

**Methods:**

A prospective multicentre cohort study was performed using patients admitted to 254 UK hospitals with COVID-19 between 17 January 2020 and 5 December 2020.

**Results:**

Of 85 687 patients, 2198 (2.6%) received acute kidney replacement therapy (KRT). Of 41 294 patients with biochemistry data, 13 000 (31.5%) had biochemical AKI: 8562 stage 1 (65.9%), 2609 stage 2 (20.1%) and 1829 stage 3 (14.1%). The main risk factors for KRT were chronic kidney disease (CKD) [adjusted odds ratio (aOR) 3.41: 95% confidence interval 3.06–3.81], male sex (aOR 2.43: 2.18–2.71) and Black race (aOR 2.17: 1.79–2.63). The main risk factors for biochemical AKI were admission respiratory rate >30 breaths per minute (aOR 1.68: 1.56–1.81), CKD (aOR 1.66: 1.57–1.76) and Black race (aOR 1.44: 1.28–1.61). There was a gradated rise in the risk of 28-day mortality by increasing severity of AKI: stage 1 aOR 1.58 (1.49–1.67), stage 2 aOR 2.41 (2.20–2.64), stage 3 aOR 3.50 (3.14–3.91) and KRT aOR 3.06 (2.75–3.39). AKI rates peaked in April 2020 and the subsequent fall in rates could not be explained by the use of dexamethasone or remdesivir.

**Conclusions:**

AKI is common in adults hospitalized with COVID-19 and it is associated with a heightened risk of mortality. Although the rates of AKI have fallen from the early months of the pandemic, high-risk patients should have their kidney function and fluid status monitored closely.

## Graphical Abstract

**Graphical Abstract:**
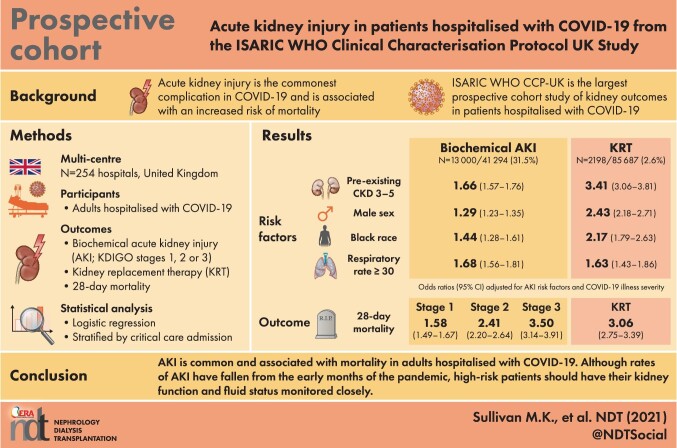

KEY LEARNING POINTS **What is already known about this subject**?

- Acute kidney injury (AKI) is the most common complication in COVID-19 and it is associated with an increased risk of mortality.
- Studies from early in the pandemic identified risk factors for COVID-AKI: male sex, older age, Black race, diabetes, chronic kidney disease (CKD), hypertension, heart disease and obesity.
- This is the largest prospective cohort study of kidney outcomes in patients hospitalized with COVID-19 with data over the course of 2020 and it includes valuable information on illness severity, race and COVID-19 specific medications.

**What this study adds**?

- Patients from minority ethnic backgrounds are at heightened risk of COVID-AKI and comorbidities like diabetes and CKD play important roles in their risk profiles.
- COVID-AKI has become less common since the first wave of the pandemic, but this is not linked to the use of dexamethasone or remdesivir.

**What impact this may have on practice or policy**?

- Although the rates of COVID-AKI have fallen from the first wave of the pandemic, it remains common, particularly in patients with CKD, patients with severe COVID-19 illness and patients of Black race.
- Given the link between COVID-AKI and mortality, clinicians should monitor the fluid balance and kidney function of patients with COVID-19 and intervene early if AKI occurs.

## INTRODUCTION

The severe acute respiratory syndrome coronavirus 2 (SARS-CoV-2) has had a major impact on global health. Although coronavirus disease 2019 (COVID-19) produces primarily pulmonary damage (acute respiratory distress syndrome—ARDS), acute kidney injury (AKI) is common [1], ranging from minor biochemical changes in serum creatinine to requirement for kidney replacement therapy (KRT: dialysis or haemofiltration).

As infection rates accelerated in New York in March 2020, there were reports of AKI in 37% of hospitalized patients [2–4], substantially higher than reports from China (<5%) [5, 6]. Given that KRT resources are finite, additional strategies were planned in some areas [7], including acute peritoneal dialysis [8]. However, AKI rates among patients with COVID-19 have fallen as the pandemic has unfolded, perhaps due to improvements in treatment, changes in practice or some other factors. Several mechanisms of AKI in COVID-19 have been postulated, including systemic inflammation [9], kidney tropism and direct damage[10, 11], collapsing glomerulopathy [12], complement activation [13] and organ crosstalk, although it seems likely from case series that acute tubular necrosis is the predominant renal pathology [14, 15]. AKI is common in all patients treated in critical care environments, so it may be that AKI in COVID-19 is merely an indicator of severe illness.

Studies of AKI from the early months of the pandemic have not been verified and updated via comprehensive studies. The International Severe Acute Respiratory and Emerging Infections Consortium (ISARIC) World Health Organization (WHO) Clinical Characterisation Protocol UK (CCP-UK) for Severe Emerging Infections was planned in 2012 to capture clinical information on any emerging infectious disease. It was activated in the UK on 17 January 2020 in response to the COVID-19 pandemic and has collected data since. It is one of the largest global cohorts of patients hospitalized with COVID-19 and it has demonstrated that renal complications are more frequent than in any other body system [16]. This study investigates AKI in detail, refining the estimates of risk factors and mortality and focusing on the potential relationships between AKI and race, illness severity and pharmaceutical intervention.

## MATERIALS AND METHODS

### Study design and patients

Adults over the age of 18 years hospitalized between 17 January 2020 and 5 December 2020 with confirmed or highly suspected SARS-CoV-2 infection leading to COVID-19 were recruited at 254 sites in England, Scotland and Wales. Data were entered into a standardised Research Electronic Data Capture secure online database [17]. Study information and materials are available online [18]. Confirmation of SARS-CoV-2 was performed using reverse-transcriptase polymerase chain reaction. Highly suspected cases were eligible for inclusion because SARS-CoV-2 was an emergent pathogen at the time of protocol activation. Exclusion criteria were long-term dialysis, nosocomial infection and readmission to hospital (i.e. only the first admission was included for each patient) [19]. Two analyses were performed:

- KRT analysis: patients with information on the need for acute KRT were included.
- Biochemical AKI analysis: patients with two or more serum creatinine results were included.

### Outcomes

The primary outcome was the use of acute KRT. The secondary outcome was biochemical AKI. We used biochemical AKI definitions based on the National Health Service AKI e-alert algorithms [20] and AKI severity was graded using Kidney Disease: Improving Global Outcomes (KDIGO) stages [21]:

- Stage 1

○ Serum creatinine >26 µmol/L higher than the lowest creatinine within 48 h
○ Serum creatinine ≥1.5–1.9 times higher than the lowest creatinine within 7 days
○ Serum creatinine ≥1.5–1.9 times higher than the median of all creatinine values 8–365 days ago

- Stage 2

○ Serum creatinine ≥2–2.9 times higher than the lowest creatinine within 7 days
○ Serum creatinine ≥2–2.9 times higher than the median of all creatinine values 8–365 days ago

- Stage 3 Biochemical

○ Serum creatinine ≥3 times higher than the lowest creatinine within 7 days
○ Serum creatinine ≥3 times higher than the median of all creatinine values 8–365 days ago

### Covariates

Race was categorized as White, Black, South Asian, East Asian and other. Socioeconomic deprivation was quantified using Index of Multiple Deprivation (IMD) scores. Smoking status was categorized as ‘Never’, ‘Previous’ and ‘Current’. Health conditions and long-term use of medications before admission were captured from available health care records by research nurses and volunteer medical students. Illness severity on admission was estimated using oxygen saturation on air and respiratory rate.

### Statistical methods

Patient characteristics were described for those who received and did not receive KRT, for those with each stage of biochemical AKI and for those from the overall cohort with and without biochemistry data. Medians and interquartile ranges (IQR) were used to describe continuous variables and percentages for categorical variables.

Logistic regression was performed to study the associations between risk factors and KRT, biochemical AKI and each stage of AKI. Adjustments were made for age, sex, race, diabetes, heart disease, chronic kidney disease (CKD), use of renin–angiotensin system blockers (RAS blockers) before admission and socioeconomic deprivation status (as these variables have previously been associated with AKI [21]), oxygen saturation on air and respiratory rate on admission (as indicators of illness severity, both as continuous variables). Age as a confounder was treated as a continuous variable and as a risk factor as a categorical variable. The missingness patterns of race, deprivation, diabetes, heart disease, CKD, admission respiratory rate and oxygen saturations were explored, and these variables were found to be missing at random. Multiple imputation using chained equations [22] was used for these variables using 10 sets, each with 10 iterations, and Rubin's rules were used to combine the results [23]. Complete case sensitivity analysis was performed and the results were compared with those from multiple imputation. Prespecified interaction analyses were performed for the relationship between race and each of KRT and biochemical AKI and considered significant if P-values were <0.01.

The relationship between AKI and 28-day mortality was described using a Kaplan–Meier survival curve. Follow-up started on the date of symptom onset or—where this was not available—the date of hospitalization. Follow-up ended on the date of death or discharge (whichever occurred first), or 28 days following hospitalization if neither event occurred. Patients were categorized by the highest stage of AKI they reached. Logistic regression was performed for 28-day mortality using the same confounders and multiple imputation approach as in the AKI analyses. These analyses were stratified by AKI stage and critical care status.

AKI rates in each month in 2020 were compared by calculating the proportion of patients with each stage of AKI. 95% confidence intervals were calculated using Wilson Score Intervals [24]. The severity of COVID-19 illness was compared using median admission 4C Mortality Scores per month [25].

The median number of days from both symptom onset and hospitalization to identification of AKI was compared per month. The proportion of patients whose AKI had resolved by the end of follow-up was calculated.

The risk of AKI in patients receiving dexamethasone was compared with patients not receiving dexamethasone using propensity score matching. Propensity score matching was used for this part of the study as a method for evaluating treatment effects using observational data [26]. Only patients receiving supplemental oxygen and admitted to the hospital after 31 May 2020 were included because dexamethasone became the standard of care for patients with COVID-19 requiring oxygen from June 2020 onwards [27]. Patients with AKI on the day of admission were excluded from this part of the analysis because the influence of dexamethasone on AKI could not be determined for them. Exact matching was performed for the month of admission with nearest neighbour matching for age, sex, race, IMD deprivation quintile, diabetes, heart disease, CKD, RAS blockers, and oxygen saturations on air and respiratory rate on admission. The same analysis was performed for remdesivir, but, in addition, patients needed satisfactory kidney and liver function on admission to be included, based on the UK prescribing guidelines for remdesivir (estimated glomerular filtration rate greater than 30 mL/min/1.73 m^2^ and alanine aminotransferase less than five times the upper limit of normal). The characteristics of the patients receiving dexamethasone and/or remdesivir were compared with those not receiving the medications. Analyses were not performed for tocilizumab because insufficient patients in the cohort received the drug.

Statistical analyses were performed using R version 3.6.1 (R Foundation for Statistical Computing, Vienna, Austria): *tidyverse, finalfit, survival, survminer, nephro, mice, MatchIt* and *forestplot* packages.

## RESULTS

Of 114 131 patients with data available at the time of the analysis, 85 687 were studied in the KRT analysis and 41 294 in the biochemical AKI analysis (Supplementary data, Figure S1). A total of 2198 (2.6%) received acute KRT, and 13 000 (31.5%) had biochemical AKI: 8562 stage 1 (65.9%), 2609 stage 2 (20.1%) and 1829 stage 3 (14.1%).

### Patient characteristics

Patient characteristics are presented by KRT status (Table 1) and stage of biochemical AKI (Table 2). Of the 85 687 patients in the KRT analysis, 63 021 (73.5%) had confirmed infection and 22 666 (26.5%) had highly suspected infection. The characteristics of patients with biochemistry data were slightly different to patients without biochemistry data (Supplementray data, Table S1). A number of comorbidities were more common in patients with biochemistry data compared with those without biochemistry data, including diabetes (24.7% versus 20.3%), CKD (17.5% versus 16.2%) and obesity (14.2% versus 10.9%).

**Table 1.** Patient characteristics by kidney replacement therapy status

		No KRT (*N* = 83 489)	KRT (*N* = 2198)
Age (years)	Median (IQR)	74 (58 to 83)	62 (54 to 70)
Sex (%) Not specified: 202 (0.2%)	Female	37 339 (44.7)	528 (24.0)
	Male	45 954 (55.0)	1664 (75.7)
Race (%) Missing values: 9718 (11.3%)	White	61 266 (82.7)	1204 (63.8)
	Black	2642 (3.6)	179 (9.5)
	South Asian	4407 (5.9)	245 (13.0)
	East Asian	543 (0.7)	25 (1.3)
	Other	5224 (7.1)	234 (12.4)
IMD quintile (%) Missing values: 3183 (3.7%)	1	16 228 (20.2)	436 (20.7)
	2	16 610 (20.7)	457 (21.7)
	3	15 836 (19.7)	362 (17.2)
	4	15 988 (19.9)	414 (19.7)
	5	15 739 (19.6)	434 (20.6)
Smoking (%) Missing values: 34 413 (40.2%)	Never	27 733 (55.7)	899 (61.0)
	Current	4390 (8.8)	86 (5.8)
	Former	17 678 (35.5)	488 (33.1)
Hypertension (%) Missing values: 8990 (10.5%)		40 304 (53.9)	1164 (62.1)
Diabetes (%) Missing values: 6952 (8.1%)		16 753 (21.8)	742 (36.9)
Chronic kidney disease (%) Missing values: 3991 (4.7%)		12 944 (16.3)	648 (31.1)
Heart disease (%) Missing values: 3601 (4.2%)		25 507 (31.9)	485 (23.6)
Lung disease (not asthma) (%) Missing values: 3767 (4.4%)		13 808 (17.3)	175 (8.5)
Asthma (%) Missing values: 3954 (4.6%)		10 848 (13.6)	306 (14.9)
Chronic liver disease (%) Missing values: 4479 (5.2%)		2659 (3.4)	67 (3.3)
Neurological disease (%) Missing values: 4274 (5.0%)		9917 (12.5)	117 (5.7)
Cancer (%) Missing values: 4412 (5.1%)		8095 (10.2)	118 (5.8)
Haematological disease (%) Missing values: 4442 (5.2%)		3435 (4.3)	86 (4.2)
Human immunodeficiency virus (%) Missing values: 5689 (6.6%)		288 (0.4)	23 (1.1)
Obesity (%) Missing values: 12 413 (14.5%)		8602 (12.1)	510 (26.6)
Rheumatological disease (%) Missing values: 4650 (5.4%)		9247 (11.7)	153 (7.6)
Dementia (%) Missing values: 4242 (5.0%)		12 386 (15.6)	23 (1.1)
RAS blockers (%) Missing values: 9926 (11.6%)		20 603 (27.9)	618 (33.6)
Calcium channel blockers (%) Missing values: 9926 (11.6%)		15 794 (21.4)	594 (32.3)
Beta-blockers (%) Missing values: 9926 (11.6%)		22 349 (30.2)	602 (32.8)
Diuretics (%) Missing values: 9926 (11.6%)		18 165 (24.6)	383 (20.8)
Statins (%) Missing values: 9926 (11.6%)		30 843 (41.7)	858 (46.7)
Systemic corticosteroids (%) Missing values: 9926 (11.6%)		8368 (11.3)	230 (12.5)
Immunosuppressants (%) Missing values: 9926 (11.6%)		1871 (2.5)	84 (4.6)
Proton pump inhibitors (%) Missing values: 9926 (11.6%)		32 347 (43.8)	771 (41.9)
Nonsteroidal anti-inflammatory drugs (%) Missing values: 9926 (11.6%)		2710 (3.7)	74 (4.0)
Aspirin (%) Missing values: 9926 (11.6%)		22 254 (30.1)	586 (31.9)
Any supplemental oxygen (%) Missing values: 281 (0.3%)		60 640 (72.9)	2091 (95.4)
Any critical care admission (%) Missing values: 47 (0.1%)		10 603 (12.7)	1752 (79.7)
Any invasive ventilation (%) Missing values: 39 (0.0%)		4872 (5.8)	1613 (73.5)
Any non-invasive ventilation (%) Missing values: 242 (0.3%)		11 709 (14.1)	1040 (47.5)

All medications were those in use before hospitalization.

**Table 2.** Patient characteristics by biochemical acute kidney injury stage

		No AKI (*N* = 28 294)	Stage 1 (*N* = 8562)	Stage 2 (*N* = 2609)	Stage 3 (*N* = 1829)
Age (years)	Median (IQR)	73 (58–83)	73 (61–83)	71 (60–80)	65 (57–75)
Sex (%) Not specified 151 (0.4%)	Female	12 105 (42.8)	3085 (36.0)	960 (36.8)	586 (32.0)
	Male	16 096 (56.9)	5441 (63.5)	1637 (62.7)	1233 (67.4)
Race (%) Missing values: 4543 (11.0%)	White	20 689 (82.2)	6183 (80.6)	1800 (78.3)	1147 (70.9)
	Black	953 (3.8)	395 (5.1)	134 (5.8)	125 (7.7)
	South Asian	1315 (5.2)	421 (5.5)	137 (6.0)	136 (8.4)
	East Asian	214 (0.9)	68 (0.9)	16 (0.7)	20 (1.2)
	Other	1989 (7.9)	608 (7.9)	211 (9.2)	190 (11.7)
IMD quintile (%) Missing values: 1734 (4.2%)	1	5022 (18.5)	1616 (19.7)	487 (19.5)	348 (19.9)
	2	5245 (19.3)	1672 (20.4)	569 (22.8)	373 (21.4)
	3	5294 (19.5)	1610 (19.7)	510 (20.4)	319 (18.3)
	4	5470 (20.2)	1605 (19.6)	448 (17.9)	324 (18.5)
	5	6092 (22.5)	1689 (20.6)	484 (19.4)	383 (21.9)
Smoking (%) Missing values: 15 327 (37.1%)	Never	9972 (55.9)	2828 (53.2)	862 (54.9)	726 (58.3)
	Current	1485 (8.3)	414 (7.8)	115 (7.3)	83 (6.7)
	Former	6381 (35.8)	2073 (39.0)	592 (37.7)	436 (35.0)
Hypertension (%) Missing values: 3620 (8.8%)		13 681 (53.1)	4795 (60.4)	1428 (59.8)	941 (59.0)
Diabetes (%) Missing values: 3378 (8.2%)		5872 (22.6)	2261 (28.9)	702 (29.7)	510 (29.9)
Chronic kidney disease (%) Missing values: 2273 (5.5%)		4040 (15.1)	2043 (25.2)	487 (19.9)	240 (13.9)
Heart disease (%) Missing values: 1997 (4.8%)		8367 (31.0)	2805 (34.4)	714 (29.2)	381 (22.0)
Lung disease (not asthma) (%) Missing values: 2164 (5.2%)		4706 (17.5)	1471 (18.1)	416 (17.0)	197 (11.4)
Asthma (%) Missing values: 2285 (5.5%)		3919 (14.6)	988 (12.2)	321 (13.1)	237 (13.7)
Chronic liver disease (%) Missing values: 2583 (6.3%)		1002 (3.8)	282 (3.5)	90 (3.7)	48 (2.8)
Neurological disease (%) Missing values: 2476 (6.0%)		3095 (11.6)	948 (11.8)	259 (10.7)	137 (8.0)
Cancer (%) Missing values: 2523 (6.1%)		2812 (10.6)	835 (10.4)	246 (10.1)	120 (7.0)
Haematological disease (%) Missing values: 2559 (6.2%)		1219 (4.6)	396 (4.9)	112 (4.6)	46 (2.7)
Human immunodeficiency virus (%) Missing values: 3190 (7.7%)		102 (0.4)	36 (0.5)	<15 (0.3)	<15 (0.9)
Obesity (%) Missing values: 6081 (14.7%)		3183 (13.2)	1086 (14.8)	408 (18.5)	348 (21.7)
Rheumatological disease (%) Missing values: 2660 (6.4%)		3163 (11.9)	878 (11.0)	258 (10.7)	141 (8.2)
Dementia (%) Missing values: 2395 (5.8%)		3498 (13.1)	1234 (15.3)	328 (13.5)	136 (7.9)
RAS-blockers (%) Missing values: 3946 (9.6%)		6895 (27.0)	2529 (32.2)	830 (35.0)	542 (34.3)
Calcium channel blockers (%) Missing values: 3946 (9.6%)		5495 (21.5)	2003 (25.5)	665 (28.1)	433 (27.4)
Beta-blockers (%) Missing values: 3946 (9.6%)		7457 (29.2)	2666 (34.0)	773 (32.6)	433 (27.4)
Diuretics (%) Missing values: 3946 (9.6%)		6079 (23.8)	2201 (28.1)	599 (25.3)	336 (21.2)
Statins (%) Missing values: 3946 (9.6%)		10 638 (41.6)	3669 (46.8)	1057 (44.6)	666 (42.1)
Systemic corticosteroids (%) Missing values: 3946 (9.6%)		3042 (11.9)	914 (11.7)	259 (10.9)	153 (9.7)
Immunosuppressants (%) Missing values: 3946 (9.6%)		776 (3.0)	296 (3.8)	66 (2.8)	47 (3.0)
Proton pump inhibitors (%) Missing values: 3946 (9.6%)		11 349 (44.4)	3440 (43.8)	1010 (42.6)	607 (38.4)
Nonsteroidal anti-inflammatory drugs (%) Missing values: 3946 (9.6%)		894 (3.5)	235 (3.0)	91 (3.8)	89 (5.6)
Aspirin (%) Missing values: 3946 (9.6%)		7400 (29.0)	2618 (33.4)	734 (31.0)	386 (24.4)
Any supplemental oxygen (%) Missing values: 402 (1.0%)		22 623 (80.8)	7464 (88.0)	2333 (90.0)	1696 (93.8)
Any critical care admission (%) Missing values: 163 (0.4%)		4838 (17.2)	2616 (30.7)	1240 (47.7)	1275 (70.0)
Any invasive ventilation (%) Missing values: 601 (1.5%)		2184 (7.8)	1615 (19.1)	1001 (38.8)	1147 (63.7)
Any non-invasive ventilation (%) Missing values: 686 (1.7%)		5232 (18.8)	2405 (28.5)	928 (36.0)	785 (43.7)

All medications were those in use before hospitalization.

### Clinical variables associated with KRT

**Figure 1:**
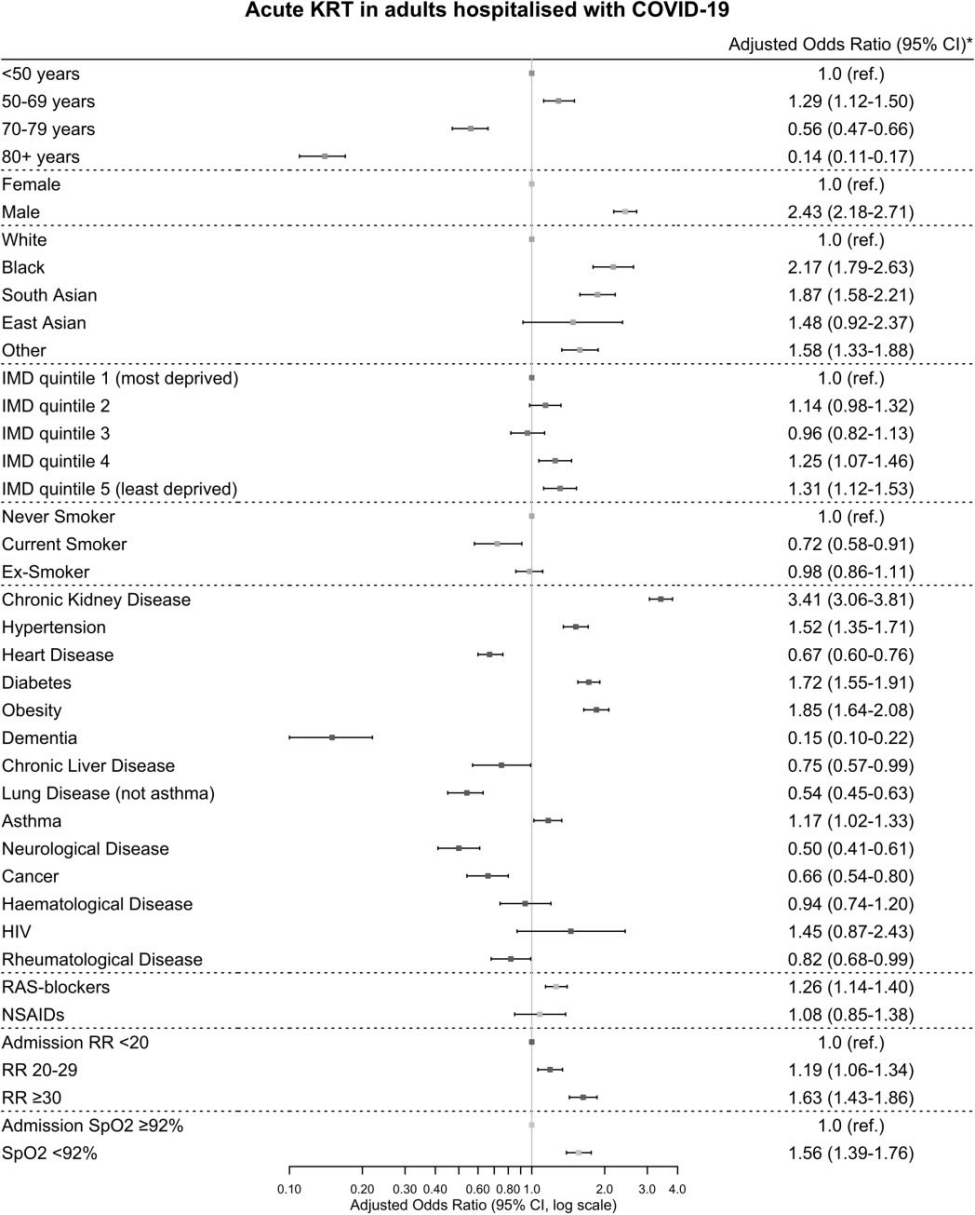
Associations between risk factors and acute kidney replacement therapy. *Adjusted for age, sex, race, deprivation quintile, chronic kidney disease, heart disease, diabetes, admission oxygen saturations on air and admission respiratory rate. HIV, human immunodeficiency virus; RR, respiratory rate; SpO2, oxygen saturations. Error bars are 95% confidence intervals (CI).

Risk factors strongly positively associated with KRT were CKD [adjusted odds ratio (aOR) 3.41: 95% confidence interval 3.06–3.81], male sex (aOR 2.43: 2.18–2.71) and Black race (aOR 2.17: 1.79–2.63) (Figure 1). Indicators of severe illness on admission associated with KRT were as follows: admission respiratory rate greater than 30 breaths per minute (aOR 1.63: 1.43–1.86) and admission oxygen saturation less than 92% on air (aOR 1.56: 1.39–1.76). Age over 80 years (aOR 0.14: 0.11–0.17) and dementia (aOR 0.15: 0.10–0.22) were negatively associated with KRT. aORs were similar for complete case sensitivity analysis (Supplementary data, Table S2).

### Clinical variables associated with biochemical AKI

**Figure 2:**
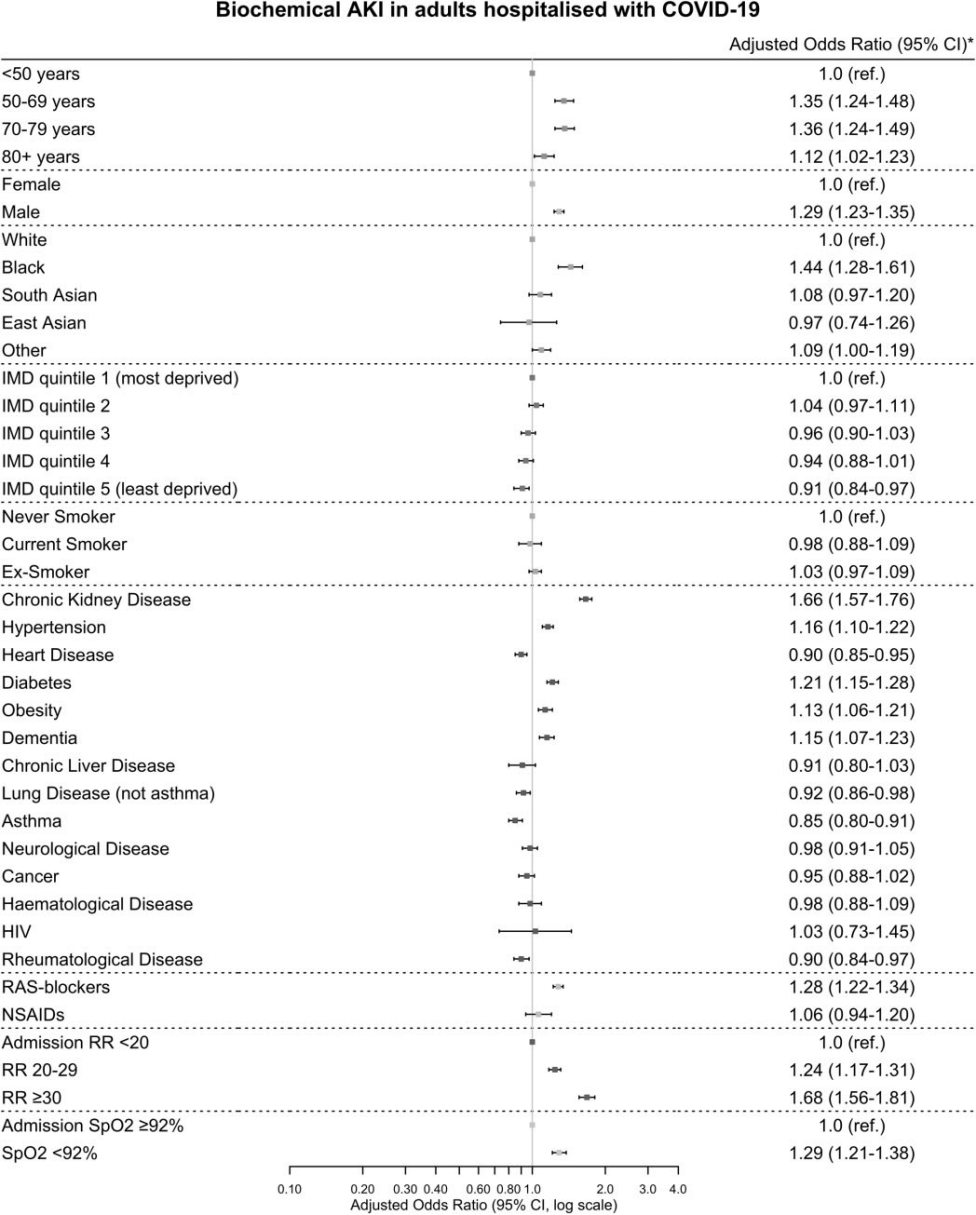
Associations between risk factors and biochemical acute kidney injury. *Adjusted for age, sex, race, deprivation quintile, chronic kidney disease, heart disease, diabetes, admission oxygen saturations on air and admission respiratory rate. HIV, human immunodeficiency virus; RR, respiratory rate; SpO2, oxygen saturations. Error bars are 95% confidence intervals (CI).

Risk factors with strongly positive associations with biochemical AKI were admission respiratory rate greater than 30 breaths per minute (aOR 1.68: 1.56–1.81), CKD (aOR 1.66: 1.57–1.76) and black race (aOR 1.44: 1.28–1.61) (Figure 2). aORs were similar for complete case sensitivity analysis (Supplementary data, Table S3). Analysis of each stage of AKI showed similar risk factors (Supplementary data, Table S4). Patients of South Asian and other race and those on non-steroidal anti-inflammatory drugs were at increased risk of stages 2 and 3 AKI, but not stage 1.

### Race analyses

For the KRT analysis, there were interactions between South Asian race and each of age, male sex, CKD and hypertension; there was an interaction between Black race and CKD; and there was an interaction between other race and each of age and CKD (P-values all <0.01) (Supplementary data, Tables S5 and S6). For the biochemical AKI analysis, there was an interaction between South Asian race and each of CKD and diabetes and there was an interaction between Black race and CKD (P-values all <0.01). Compared with White patients, those from minority race groups in the analysis were younger and proportionally more of them were admitted to critical care (Supplementary data, Table S7). CKD was more common in White patients (17.6%) than those from minority race groups: Black (15.7%), South Asian (14.5%), East Asian (9.5%) and other (12.6%).

### 28-day mortality risk

**Figure 3:**
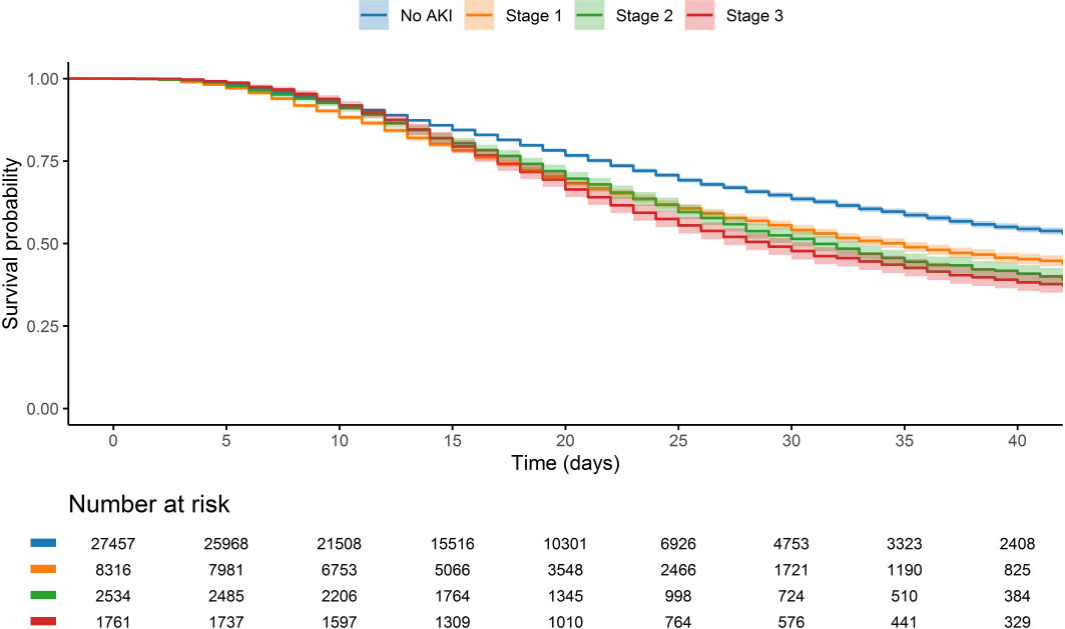
Kaplan–Meier plot of 28-day mortality by biochemical acute kidney injury status. Time is after symptom onset. Shaded area represents 95% confidence intervals.

**Figure 4:**
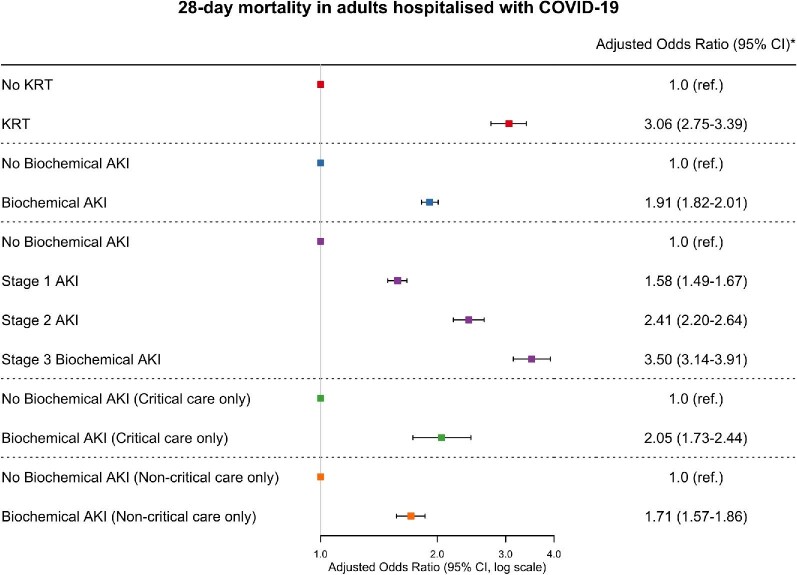
Associations between acute kidney injury and 28-day mortality. P-values for all groups <0.001. *Adjusted for age, sex, race, deprivation quintile, chronic kidney disease, heart disease, diabetes, admission oxygen saturations on air and admission respiratory rate. Error bars are 95% confidence intervals (CI).

There was an increased risk of mortality for those receiving KRT (aOR 3.06: 2.75–3.39) and those with biochemical AKI (aOR 1.91: 1.82–2.01) (Figures 3 and 4). The associations for biochemical AKI were present in patients treated within and outside critical care, and mortality risk was higher in those with stage 3 than less severe stages (aOR 3.50: 3.14–3.91).

### AKI rates by month

**Figure 5:**
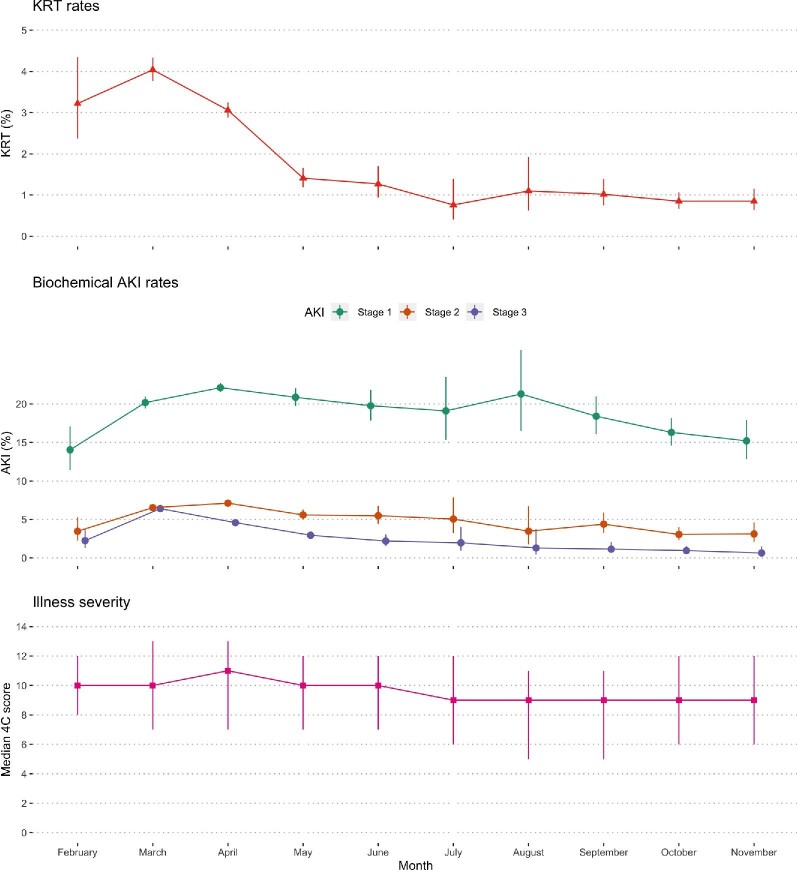
Acute kidney injury rates and 4C scores by month in 2020. Error bars represent 95% confidence intervals for KRT and biochemical AKI rates and interquartile ranges for illness severity.

KRT rates peaked in March 2020 at 4.0% and biochemical AKI in April 2020 at 33.8% (Figure 5). After June 2020, there was a marginal reduction in 4C Mortality Scores: median score 11 (IQR 7–13) in April 2020 and 9 (IQR 6–12) from July 2020 onwards.

### Timing of AKI

Amongst patients with AKI, the median time from symptom onset to AKI was 6 days (IQR 2–11). Amongst patients with AKI, 7123 of 13 000 (54.8%) had it on the day of admission and the median time from admission to AKI was 0 days (IQR 0–3). There was no trend in the timing of AKI throughout the months of 2020 (Supplementary data, Table S8). At the end of follow-up, AKI had resolved in 9758/13 000 (75.1%) of patients.

### Dexamethasone/remdesivir 

Compared with the patients not receiving these medications, those receiving dexamethasone and/or remdesivir were on average 6 years younger and had higher rates of antimicrobial use and treatment in critical care (Table S9a–d, S10 and S11). The use of dexamethasone was positively associated with KRT [odds ratio (OR) 2.23: 1.09–4.80] and there was no relationship between dexamethasone and biochemical AKI (OR 0.90: 0.51–1.56). There was no relationship between the use of remdesivir and KRT (OR 1.09: 0.38–2.72) or biochemical AKI (OR 0.84: 0.52–1.34).

## DISCUSSION

In this prospective multicentre study of up to 85 687 patients hospitalized with COVID-19, we have described risk factors and associations for COVID-19-induced AKI and related mortality. Men, patients with CKD, diabetes, hypertension and obesity, patients from minority race backgrounds and those with severe COVID-19 on admission were at highest risk of AKI related to COVID-19. All stages of AKI were associated with an increased risk of mortality and there was a graded rise in mortality risk by increasing AKI severity. Rates of AKI peaked in April 2020 and although they fell following the first wave of the pandemic, improvements in COVID-19 treatment via pharmaceutical developments were not associated with risk reductions.

The rate of AKI in our study was 31.5%, matching reports from the USA [2, 28]. The KRT rate in our study was 2.6%, lower than in some others (14–15%) [4, 29]. However, these were single-centre studies and clinical practice such as eligibility criteria for critical care treatment may have influenced KRT rates. Declines in AKI rates following the first wave of the pandemic have been reported elsewhere [28, 30], but the reasons for this have not been evaluated. Our findings suggest that improvements in the treatment of COVID-19 with dexamethasone and remdesivir did not directly account for the falls in AKI rates. By comparison, the RECOVERY randomized controlled trial found that fewer patients randomized to dexamethasone needed KRT [31]. This may be because, after the end of May 2020, these medications were given to the most unwell patients with COVID-19 in the hospital. Although we adjusted for several confounding variables including illness severity, there is likely to be residual confounding that could affect the results.

Beyond pharmaceutical developments, the management of COVID-19 patients changed significantly during 2020. National Institute for Health and Care Excellence guidelines in the UK encouraged the maintenance of euvolaemia in COVID-19 [32]. However, some clinicians employed conservative fluid resuscitation strategies in the early months of the pandemic. This approach originated from the treatment of patients with non-COVID-19 ARDS and was advocated in the COVID-19 Surviving Sepsis Guideline [33]. We postulate that conservative fluid strategies may have inadvertently contributed to the development of AKI in some patients in the early months of the pandemic, such as in those with precarious oxygenation and fluid losses. As previously reported from the ISARIC WHO CCP-UK study, the use of invasive mechanical ventilation in patients with COVID-19 fell significantly as the pandemic unfolded [34]. This change in practice may have had an additional impact on AKI. We found the risk of biochemical AKI was lower in the oldest adults (over 80 years) compared with other age groups (50–79 years). This was not associated with reduced frequency of blood tests in the oldest adults, hence the reasons for this trend are unclear. The declining rates of AKI over time may in part be due to increasing clinician awareness of AKI in COVID-19, prompting them to monitor their patients’ fluid status and blood tests more closely, as well as decreasing illness severity [34].

We have verified findings from smaller studies from the first wave of the pandemic, including the role of AKI as a risk factor for mortality in COVID-19. Overall, 40.4% of patients with biochemical AKI in our study died, which falls between the 34% [2, 28, 6] and 51.8% [35] reported in previous studies. Even patients with minor biochemical changes (stage 1 AKI) were at increased risk of dying, highlighting that all patients with COVID-19 and AKI should have targeted monitoring and optimization of their fluid status. Although we have confirmed that mortality risk rose with the increasing stage of AKI, our ORs are lower than in previous studies [6, 28, 36]. This may be because the patients in our ‘no AKI’ reference group were more co-morbid than the rest of the cohort, with high rates of diabetes and CKD.

The AKI risk factors we identified were similar to those reported previously [2, 28]. We have confirmed a particularly high risk of AKI in patients from minority race backgrounds and our findings suggest this is contributed to by comorbidities [37]. A study of 1737 patients with COVID-19 in East London (60% non-White) demonstrated an increased risk of mortalityin Black and Asian patients, independent of comorbidities [38]. Several factors are postulated to contribute to this increased risk, including a higher prevalence of comorbidities that are associated with greater COVID-19 disease severity, cultural factors, host genetics, cultural and lifestyle factors, and inequality [39]. We found that non-White patients admitted to the hospital with COVID-19 were younger and more likely to be admitted to critical care than their White counterparts. This may explain to a large extent their increased risk of requiring KRT. South Asian race was a predictor of stages 2 and 3 AKI, and our interaction analysis suggests this was contributed to by CKD and diabetes. Our results suggest that the increased risk of biochemical AKI in Black patients is contributed to by CKD, even though the proportion of Black patients with CKD was less than for White patients. It is possible that prior CKD was infrequently recorded in patients of Black race [40]. In addition to multiple socioeconomic and health risk factors associated with adverse outcomes in people of Black race in COVID-19 [41], AKI-specific risk may in some part be attributable to the possession of high-risk *APOL1* genotypes [42], which are present in people of West African ancestry. These alleles are associated with a greatly increased risk of CKD in people of Black race in North America [43] and have been implicated in COVID-19-related glomerular disease [44].

Our study has some notable strengths. To our knowledge, it is the largest study of AKI in COVID-19 to date. Our cohort comprised patients from 254 acute hospital sites spanning most of 2020, allowing us to evaluate the temporal variations in AKI and KRT rates over the first wave and part of the second wave of the pandemic. Our cohort included a significant number of patients who were prescribed dexamethasone and remdesivir, thus allowing us to assess the relationship between the use of these medications and AKI. We have explored additional crucial areas: the relationship between race and AKI and illness severity as a key risk factor for AKI.

Our study has some limitations. Some 26.5% of the patients were identified as having COVID-19 before testing for SARS-CoV-2 was universally available. Some of these patients may therefore have had illnesses other than COVID-19. In our biochemical AKI analysis, we excluded patients without two or more recorded creatinine values, risking the introduction of selection bias. Despite slightly higher rates of comorbidities such as CKD in those included in the study, the patients without biochemistry data were very similar. Some blood results during an individual's admission may not have been available if they were not recorded in the database. This could have an impact on AKI detection and accurate categorization of the AKI stage. The execution of separate KRT and biochemical AKI analyses was appropriate for two reasons. First, the use of acute KRT in an individual is considerably linked to illness severity and whether clinicians decide it is appropriate to care for them in a critical care environment or a ward (which may depend on pre-morbid health status). Second, due to the challenges of real-time data collection during a pandemic, creatinine results were available for relatively few patients and we sought to study as many patients as possible. We did not have access to baseline kidney function, therefore we may have missed some AKI events and we were unable to stratify risk by severity of baseline CKD. Although most cases of AKI had resolved by the end of in-hospital follow-up, we did not have access to kidney function following discharge and so we were unable to study long-term recovery following AKI. We did not have data on urine output or fluid resuscitation regimens, although it is difficult reliably to record this information outside of critical care settings.

In conclusion, AKI was common in patients hospitalized with COVID-19 and these patients were at high risk of death. The patients at highest risk of AKI were typically men, Black, with CKD and with severe COVID-19 illness on admission. AKI rates have fallen since the early months of the pandemic, despite no observed influence of pharmaceutical developments. This may reflect changes in attitudes towards fluid balance. Clinicians should monitor the kidney function and fluid status of patients with COVID-19 closely and intervene early if AKI develops.

## Supplementary Material

gfab303_Supplemental_File

## Data Availability

This work uses data provided by patients and collected by the NHS as part of their care and support #DataSavesLives. The CO-CIN data were collated by ISARIC4C Investigators. ISARIC4C welcomes applications for data and material access through our Independent Data and Material Access Committee (https://isaric4c.net). This research used data assets made available by National Safe Haven as part of the Data and Connectivity National Core Study, led by Health Data Research UK in partnership with the Office for National Statistics and funded by UK Research and Innovation (research which commenced between 1 October 2020 and 31 March 2021, grant Ref. MC_PC_20029; 1 April 2021 to 30 September 2022, grant Ref. MC_PC_20058).

## Notes

ISARIC4C Investigators are listed in the supplementary data: https://isaric4c.net/about/authors/ Study registration ISRCTN66726260. The ISARIC WHO CCP-UK study was registered at https://www.isrctn.com/ISRCTN66726260 and designated an Urgent Public Health Research Study by NIHR.
