# Supplementary material for: Acute kidney injury in patients hospitalized with COVID-19 from the ISARIC WHO CCP-UK Study: a prospective, multicentre cohort study
Source: Nephrol Dial Transplant. 2021 Oct 18;37(2):271–84. doi: 10.1093/ndt/gfab303 (PMC8788218; doi:10.1093/ndt/gfab303)
Supplement: gfab303_Supplemental_File [file gfab303_supplemental_file.docx]

**ISARIC4C Investigators**

Consortium Lead Investigator: J Kenneth Baillie

Chief Investigator: Malcolm G Semple

Co-Lead Investigator: Peter JM Openshaw

ISARIC Clinical Coordinator: Gail Carson

Co-Investigator: Beatrice Alex, Benjamin Bach, Wendy S Barclay, Debby Bogaert, Meera Chand, Graham S Cooke, Annemarie B Docherty, Jake Dunning, Ana da Silva Filipe, Tom Fletcher, Christoper A Green, Ewen M Harrison, Julian A Hiscox, Antonia Ying Wai Ho, Peter W Horby, Samreen Ijaz, Saye Khoo, Paul Klenerman, Andrew Law, Wei Shen Lim, Alexander J Mentzer, Laura Merson, Alison M Meynert, Mahdad Noursadeghi, Shona C Moore, Massimo Palmarini, William A Paxton, Georgios Pollakis, Nicholas Price, Andrew Rambaut, David L Robertson, Clark D Russell, Vanessa Sancho-Shimizu, Janet T Scott, Thushan de Silva, Louise Sigfrid, Tom Solomon, Shiranee Sriskandan, David Stuart, Charlotte Summers, Richard S Tedder, Emma C Thomson, AA Roger Thompson, Ryan S Thwaites, Lance CW Turtle, Maria Zambon. Project Manager: Hayley Hardwick, Chloe Donohue, Ruth Lyons, Fiona Griffiths, Wilna Oosthuyzen. Data Analyst: Lisa Norman, Riinu Pius, Thomas M Drake, Cameron J Fairfield, Stephen R Knight, Kenneth A Mclean, Derek Murphy, Catherine A Shaw. Data and Information System Manager: Jo Dalton, Michelle Girvan, Egle Saviciute, Stephanie Roberts, Janet Harrison, Laura Marsh, Marie Connor, Sophie Halpin, Clare Jackson, Carrol Gamble. Data Integration and Presentation: Gary Leeming, Andrew Law, Murray Wham, Sara Clohisey, Ross Hendry, James Scott-Brown. Material Management: William Greenhalf, Victoria Shaw, Sara McDonald. Patient Engagement: Seán Keating. Outbreak Laboratory Staff and Volunteers: Katie A. Ahmed, Jane A Armstrong, Milton Ashworth, Innocent G Asiimwe, Siddharth Bakshi, Samantha L Barlow, Laura Booth, Benjamin Brennan, Katie Bullock, Benjamin WA Catterall, Jordan J Clark, Emily A Clarke, Sarah Cole, Louise Cooper, Helen Cox, Christopher Davis, Oslem Dincarslan, Chris Dunn, Philip Dyer, Angela Elliott, Anthony Evans, Lorna Finch, Lewis WS Fisher, Terry Foster, Isabel Garcia-Dorival, William Greenhalf, Philip Gunning, Catherine Hartley, Rebecca L Jensen, Christopher B Jones, Trevor R Jones, Shadia Khandaker, Katharine King, Robyn T. Kiy, Chrysa Koukorava, Annette Lake, Suzannah Lant, Diane Latawiec, Lara Lavelle-Langham, Daniella Lefteri, Lauren Lett, Lucia A Livoti, Maria Mancini, Sarah McDonald, Laurence McEvoy, John McLauchlan, Soeren Metelmann, Nahida S Miah, Joanna Middleton, Joyce Mitchell, Shona C Moore, Ellen G Murphy, Rebekah Penrice-Randal, Jack Pilgrim, Tessa Prince, Will Reynolds, P. Matthew Ridley, Debby Sales, Victoria E Shaw, Rebecca K Shears, Benjamin Small, Krishanthi S Subramaniam, Agnieska Szemiel, Aislynn Taggart, Jolanta Tanianis-Hughes, Jordan Thomas, Erwan Trochu, Libby van Tonder, Eve Wilcock, J. Eunice Zhang, Lisa Flaherty, Nicole Maziere, Emily Cass, Alejandra Doce Carracedo, Nicola Carlucci, Anthony Holmes, Hannah Massey. Edinburgh Laboratory Staff and Volunteers: Lee Murphy, Nicola Wrobel, Sarah McCafferty, Kirstie Morrice, Alan MacLean. Local Principal Investigators: Kayode Adeniji, Daniel Agranoff, Ken Agwuh, Dhiraj Ail, Erin L. Aldera, Ana Alegria, Brian Angus, Abdul Ashish, Dougal Atkinson, Shahedal Bari, Gavin Barlow, Stella Barnass, Nicholas Barrett, Christopher Bassford, Sneha Basude, David Baxter, Michael Beadsworth, Jolanta Bernatoniene, John Berridge, Nicola Best, Pieter Bothma, David Chadwick, Robin Brittain-Long, Naomi Bulteel, Tom Burden, Andrew Burtenshaw, Vikki Caruth, David Chadwick, Duncan Chambler, Nigel Chee, Jenny Child, Srikanth Chukkambotla, Tom Clark, Paul Collini, Catherine Cosgrove, Jason Cupitt, Maria-Teresa Cutino-Moguel, Paul Dark, Chris Dawson, Samir Dervisevic, Phil Donnison, Sam Douthwaite, Ingrid DuRand, Ahilanadan Dushianthan, Tristan Dyer, Cariad Evans, Chi Eziefula, Chrisopher Fegan, Adam Finn, Duncan Fullerton, Sanjeev Garg, Sanjeev Garg, Atul Garg, Effrossyni Gkrania-Klotsas, Jo Godden, Arthur Goldsmith, Clive Graham, Elaine Hardy, Stuart Hartshorn, Daniel Harvey, Peter Havalda, Daniel B Hawcutt, Maria Hobrok, Luke Hodgson, Anil Hormis, Michael Jacobs, Susan Jain, Paul Jennings, Agilan Kaliappan, Vidya Kasipandian, Stephen Kegg, Michael Kelsey, Jason Kendall, Caroline Kerrison, Ian Kerslake, Oliver Koch, Gouri Koduri, George Koshy, Shondipon Laha, Steven Laird, Susan Larkin, Tamas Leiner, Patrick Lillie, James Limb, Vanessa Linnett, Jeff Little, Mark Lyttle, Michael MacMahon, Emily MacNaughton, Ravish Mankregod, Huw Masson, Elijah Matovu, Katherine McCullough, Ruth McEwen, Manjula Meda, Gary Mills, Jane Minton, Mariyam Mirfenderesky, Kavya Mohandas, Quen Mok, James Moon, Elinoor Moore, Patrick Morgan, Craig Morris, Katherine Mortimore, Samuel Moses, Mbiye Mpenge, Rohinton Mulla, Michael Murphy, Megan Nagel, Thapas Nagarajan, Mark Nelson, Matthew K. O'Shea, Igor Otahal, Marlies Ostermann, Mark Pais, Selva Panchatsharam, Danai Papakonstantinou, Hassan Paraiso, Brij Patel, Natalie Pattison, Justin Pepperell, Mark Peters, Mandeep Phull, Stefania Pintus, Jagtur Singh Pooni, Frank Post, David Price, Rachel Prout, Nikolas Rae, Henrik Reschreiter, Tim Reynolds, Neil Richardson, Mark Roberts, Devender Roberts, Alistair Rose, Guy Rousseau, Brendan Ryan, Taranprit Saluja, Aarti Shah, Prad Shanmuga, Anil Sharma, Anna Shawcross, Jeremy Sizer, Manu Shankar-Hari, Richard Smith, Catherine Snelson, Nick Spittle, Nikki Staines, Tom Stambach, Richard Stewart, Pradeep Subudhi, Tamas Szakmany, Kate Tatham, Jo Thomas, Chris Thompson, Robert Thompson, Ascanio Tridente, Darell Tupper-Carey, Mary Twagira, Andrew Ustianowski, Nick Vallotton, Lisa Vincent-Smith, Shico Visuvanathan, Alan Vuylsteke, Sam Waddy, Rachel Wake, Andrew Walden, Ingeborg Welters, Tony Whitehouse, Paul Whittaker, Ashley Whittington, Padmasayee Papineni, Meme Wijesinghe, Martin Williams, Lawrence Wilson, Sarah Cole, Stephen Winchester, Martin Wiselka, Adam Wolverson, Daniel G Wooton, Andrew Workman, Bryan Yates, Peter Young.

Figure S1. Consort Diagram


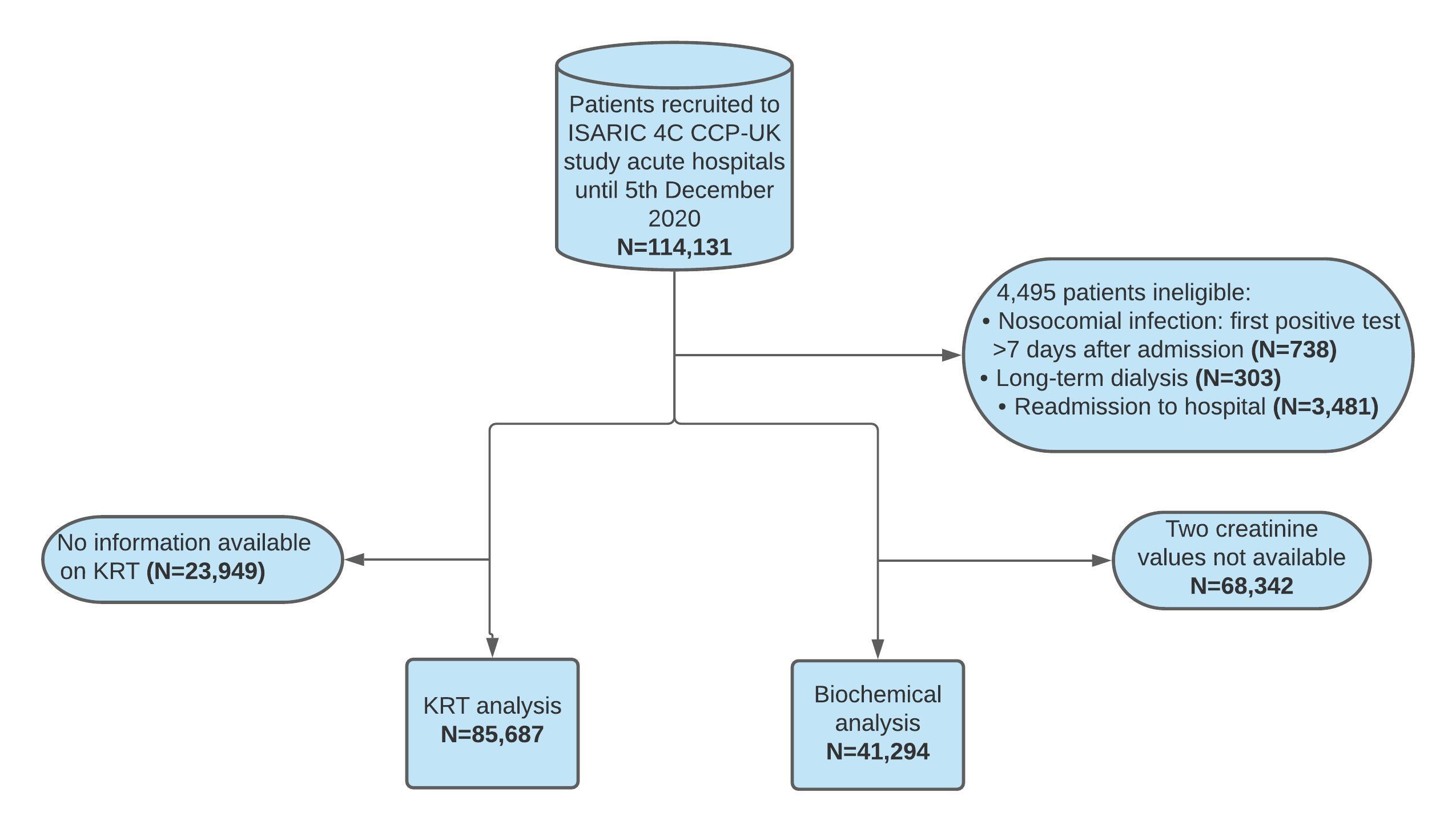


KRT, kidney replacement therapy

Table S1. Baseline characteristics by availability of biochemistry

|  | | **Biochemistry available**  **N=41294** | **No biochemistry available**  **N=68342** |
| --- | --- | --- | --- |
| Age (years) | Median (IQR) | 72 (59 to 82) | 74 (57 to 84) |
| Sex | Female | 16896 (40.7) | 29277 (46.8) |
|  | Male | 24587 (59.3) | 33251 (53.2) |
| Race | White | 30099 (81.2) | 45066 (83.5) |
|  | Black | 1613 (4.4) | 1596 (3.0) |
|  | South Asian | 2021 (5.5) | 3554 (6.6) |
|  | East Asian | 318 (0.9) | 335 (0.6) |
|  | Other | 3008 (8.1) | 3422 (6.3) |
| IMD quintile | 1 | 7508 (18.8) | 12590 (20.8) |
|  | 2 | 7920 (19.9) | 12634 (20.9) |
|  | 3 | 7812 (19.6) | 12054 (19.9) |
|  | 4 | 7933 (19.9) | 12354 (20.4) |
|  | 5 | 8716 (21.9) | 10852 (17.9) |
| Smoking | Current | 2129 (8.1) | 2882 (9.5) |
|  | Former | 9567 (36.5) | 10618 (35.0) |
|  | Never | 14487 (55.3) | 16878 (55.6) |
| Hypertension | | 21033 (55.3) | 28646 (53.9) |
| Diabetes | | 9423 (24.7) | 9968 (20.3) |
| Chronic kidney disease | | 6893 (17.5) | 8329 (16.2) |
| Heart disease | | 12404 (31.3) | 16589 (32.1) |
| Lung disease (not asthma) | | 6867 (17.4) | 8879 (17.2) |
| Asthma | | 5520 (14.0) | 6999 (13.6) |
| Chronic liver disease | | 1439 (3.7) | 1642 (3.2) |
| Neurological disease | | 4501 (11.5) | 6621 (12.9) |
| Cancer | | 4058 (10.4) | 5121 (10.0) |
| Haematological disease | | 1789 (4.6) | 2044 (4.0) |
| Human immunodeficiency virus | | 161 (0.4) | 185 (0.4) |
| Obesity | | 5053 (14.2) | 4964 (10.9) |
| Rheumatological disease | | 4497 (11.5) | 5976 (11.7) |
| Dementia | | 5263 (13.4) | 8549 (16.7) |
| RAS-blockers | | 10885 (28.9) | 14489 (27.8) |
| Calcium channel blockers | | 8655 (23.0) | 10897 (20.9) |
| Beta-blockers | | 11447 (30.4) | 15958 (30.6) |
| Diuretics | | 9318 (24.7) | 12773 (24.5) |
| Statins | | 16165 (42.9) | 21707 (41.6) |
| Systemic corticosteroids | | 4403 (11.7) | 5810 (11.1) |
| Immunosuppressants | | 1191 (3.2) | 1137 (2.2) |
| Proton pump inhibitors | | 16563 (44.0) | 23007 (44.1) |
| Non-steroidal anti-inflammatory drugs | | 1319 (3.5) | 2046 (3.9) |
| Aspirin | | 11254 (29.9) | 15982 (30.6) |

Table S2. Complete case sensitivity analysis for kidney replacement therapy

|  | | **Odds ratio**  **(univariable)** | **Odds ratio***  **(multivariable)** |
| --- | --- | --- | --- |
| Race | White | - | - |
|  | Black | 3.45 (2.92-4.04, p<0.001) | 1.83 (1.31-2.52, p<0.001) |
|  | South Asian | 2.83 (2.45-3.25, p<0.001) | 2.14 (1.64-2.76, p<0.001) |
|  | East Asian | 2.34 (1.52-3.43, p<0.001) | 1.42 (0.59-2.87, p=0.377) |
|  | Other | 2.28 (1.97-2.62, p<0.001) | 1.29 (0.94-1.72, p=0.101) |
| Sex | Male | 2.56 (2.32-2.83, p<0.001) | 2.35 (1.96-2.84, p<0.001) |
| IMD quintile | 1 | - | - |
|  | 2 | 1.02 (0.90-1.17, p=0.726) | 1.10 (0.86-1.41, p=0.453) |
|  | 3 | 0.85 (0.74-0.98, p=0.025) | 0.85 (0.65-1.11, p=0.243) |
|  | 4 | 0.96 (0.84-1.10, p=0.596) | 1.15 (0.89-1.48, p=0.299) |
|  | 5 | 1.03 (0.90-1.17, p=0.705) | 1.37 (1.07-1.77, p=0.014) |
| Age (years) | <50 | - | - |
|  | 50-69 | 1.85 (1.64-2.10, p<0.001) | 1.17 (0.92-1.49, p=0.201) |
|  | 70-79 | 0.80 (0.69-0.92, p=0.002) | 0.44 (0.33-0.59, p<0.001) |
|  | 80+ | 0.14 (0.12-0.18, p<0.001) | 0.09 (0.07-0.13, p<0.001) |
| Respiratory rate** | <20 | - | - |
|  | 20-29 | 1.44 (1.30-1.60, p<0.001) | 1.04 (0.85-1.25, p=0.719) |
|  | >30 | 2.54 (2.27-2.83, p<0.001) | 1.33 (1.05-1.66, p=0.015) |
| Oxygen saturation (%)** | >=92 | - | - |
|  | <92 | 3.00 (2.64-3.42, p<0.001) | 2.54 (2.11-3.04, p<0.001) |
| Smoking | Never | - | - |
|  | Current | 0.60 (0.48-0.75, p<0.001) | 0.46 (0.27-0.72, p=0.001) |
|  | Former | 0.85 (0.76-0.95, p=0.005) | 0.84 (0.66-1.05, p=0.125) |
| Chronic kidney disease | | 2.33 (2.12-2.56, p<0.001) | 4.98 (4.15-5.98, p<0.001) |
| Heart disease | | 0.66 (0.60-0.73, p<0.001) | 0.82 (0.67-0.99, p=0.039) |
| Diabetes | | 2.09 (1.91-2.29, p<0.001) | 1.79 (1.50-2.13, p<0.001) |
| Hypertension | | 1.40 (1.28-1.54, p<0.001) | 1.78 (1.45-2.18, p<0.001) |
| Chronic liver disease | | 0.98 (0.76-1.25, p=0.883) | 0.96 (0.64-1.40, p=0.845) |
| Lung disease (not asthma) | | 0.45 (0.38-0.52, p<0.001) | 0.64 (0.49-0.83, p=0.001) |
| Asthma | | 1.11 (0.98-1.25, p=0.096) | 1.24 (0.98-1.54, p=0.065) |
| Neurological disease | | 0.43 (0.35-0.51, p<0.001) | 0.47 (0.33-0.65, p<0.001) |
| Cancer | | 0.54 (0.45-0.65, p<0.001) | 0.56 (0.39-0.78, p=0.001) |
| Haematological disease | | 0.97 (0.77-1.20, p=0.791) | 0.87 (0.58-1.27, p=0.504) |
| Human immunodeficiency virus | | 3.11 (1.97-4.66, p<0.001) | 1.97 (0.79-4.18, p=0.104) |
| Obesity | | 2.64 (2.38-2.93, p<0.001) | 1.99 (1.62-2.45, p<0.001) |
| Rheumatological disease | | 0.62 (0.52-0.73, p<0.001) | 0.68 (0.49-0.92, p=0.017) |
| Dementia | | 0.06 (0.04-0.09, p<0.001) | 0.11 (0.05-0.21, p<0.001) |
| RAS-blockers | | 1.31 (1.19-1.45, p<0.001) | 1.13 (0.94-1.36, p=0.184) |
| Non-steroidal anti-inflammatory drugs | | 1.10 (0.86-1.38, p=0.418) | 0.67 (0.39-1.09, p=0.133) |

*Adjusted for age, sex, race, deprivation quintile, chronic kidney disease, heart disease, diabetes, admission oxygen saturations on air and admission respiratory rate.

**On admission

Table S3. Complete case sensitivity analysis for biochemical acute kidney injury

|  | | **Odds Ratio**  **(univariable)** | **Odds Ratio***  **(multivariable)** |
| --- | --- | --- | --- |
| Race | White | - | - |
|  | Black | 1.56 (1.40-1.72, p<0.001) | 1.58 (1.34-1.87, p<0.001) |
|  | South Asian | 1.20 (1.09-1.31, p<0.001) | 1.04 (0.89-1.22, p=0.602) |
|  | East Asian | 1.10 (0.87-1.39, p=0.422) | 0.91 (0.61-1.33, p=0.618) |
|  | Other | 1.15 (1.06-1.24, p=0.001) | 1.10 (0.96-1.26, p=0.155) |
| Sex | Male | 1.35 (1.29-1.41, p<0.001) | 1.27 (1.18-1.36, p<0.001) |
| IMD quintile | 1 | - | - |
|  | 2 | 1.02 (0.95-1.09, p=0.542) | 0.97 (0.87-1.08, p=0.560) |
|  | 3 | 0.94 (0.88-1.01, p=0.097) | 0.98 (0.88-1.09, p=0.751) |
|  | 4 | 0.89 (0.83-0.95, p=0.001) | 0.88 (0.79-0.98, p=0.017) |
|  | 5 | 0.86 (0.80-0.92, p<0.001) | 0.91 (0.82-1.01, p=0.083) |
| Age (years) | <50 | - | - |
|  | 50-69 | 1.60 (1.48-1.72, p<0.001) | 1.46 (1.28-1.67, p<0.001) |
|  | 70-79 | 1.58 (1.46-1.71, p<0.001) | 1.56 (1.35-1.79, p<0.001) |
|  | 80+ | 1.23 (1.14-1.33, p<0.001) | 1.34 (1.16-1.54, p<0.001) |
| Respiratory rate** | <20 | - | - |
|  | 20-29 | 1.29 (1.23-1.35, p<0.001) | 1.13 (1.05-1.22, p=0.001) |
|  | >30 | 1.87 (1.76-1.98, p<0.001) | 1.39 (1.25-1.55, p<0.001) |
| Oxygen saturation (%)** | >=92 | - | - |
|  | <92 | 1.71 (1.61-1.82, p<0.001) | 1.48 (1.37-1.61, p<0.001) |
| Smoking | Never | - | - |
|  | Current | 0.93 (0.84-1.03, p=0.161) | 1.00 (0.85-1.18, p=0.982) |
|  | Former | 1.10 (1.04-1.16, p=0.001) | 1.04 (0.95-1.15, p=0.382) |
| Chronic kidney disease | | 1.63 (1.55-1.73, p<0.001) | 1.80 (1.66-1.96, p<0.001) |
| Heart disease | | 1.03 (0.98-1.08, p=0.258) | 0.95 (0.88-1.03, p=0.217) |
| Diabetes | | 1.41 (1.35-1.49, p<0.001) | 1.25 (1.16-1.35, p<0.001) |
| Hypertension | | 1.33 (1.27-1.39, p<0.001) | 1.12 (1.04-1.21, p=0.004) |
| Chronic liver disease | | 0.91 (0.81-1.02, p=0.121) | 1.11 (0.94-1.30, p=0.228) |
| Lung disease (not asthma) | | 0.96 (0.91-1.01, p=0.138) | 0.92 (0.84-1.00, p=0.064) |
| Asthma | | 0.84 (0.79-0.90, p<0.001) | 0.83 (0.75-0.92, p<0.001) |
| Neurological disease | | 0.94 (0.88-1.01, p=0.083) | 0.92 (0.83-1.02, p=0.118) |
| Cancer | | 0.92 (0.86-0.99, p=0.031) | 1.04 (0.93-1.15, p=0.517) |
| Haematological disease | | 0.99 (0.89-1.10, p=0.877) | 1.04 (0.89-1.21, p=0.601) |
| Human immunodeficiency virus | | 1.26 (0.91-1.73, p=0.159) | 1.12 (0.65-1.88, p=0.662) |
| Obesity | | 1.30 (1.22-1.38, p<0.001) | 1.02 (0.91-1.14, p=0.726) |
| Rheumatological disease | | 0.87 (0.81-0.93, p<0.001) | 0.89 (0.80-0.98, p=0.025) |
| Dementia | | 1.07 (1.00-1.13, p=0.043) | 1.20 (1.09-1.32, p<0.001) |
| RAS-blockers | | 1.34 (1.27-1.40, p<0.001) | 1.29 (1.20-1.39, p<0.001) |
| Nonsteroidal anti-inflammatory drugs | | 1.01 (0.89-1.13, p=0.926) | 0.88 (0.72-1.07, p=0.200) |

*Adjusted for age, sex, race, deprivation quintile, chronic kidney disease, heart disease, diabetes, admission oxygen saturations on air and admission respiratory rate.

**On admission

Table S4. Associations between risk factors and each stage of acute kidney injury

|  | | **Stage 1. Odds ratio*** | **Stage 2. Odds Ratio*** | **Stage 3. Odds Ratio*** |
| --- | --- | --- | --- | --- |
| Race | White | NA | NA | NA |
|  | Black | 1.44 (1.28-1.61, p<0.001) | 1.49 (1.28-1.74, p<0.001) | 1.64 (1.32-2.02, p<0.001) |
|  | South Asian | 1.08 (0.97-1.20, p=0.168) | 1.18 (1.02-1.36, p=0.029) | 1.37 (1.12-1.68, p=0.003) |
|  | East Asian | 0.97 (0.74-1.26, p=0.807) | 0.84 (0.56-1.28, p=0.422) | 1.12 (0.65-1.95, p=0.676) |
|  | Other | 1.09 (1.00-1.19, p=0.061) | 1.17 (1.03-1.32, p=0.016) | 1.26 (1.06-1.51, p=0.011) |
| Sex | Male | 1.29 (1.23-1.35, p<0.001) | 1.23 (1.15-1.32, p<0.001) | 1.34 (1.21-1.50, p<0.001) |
| IMD quintile | 1 | NA | NA | NA |
|  | 2 | 1.04 (0.97-1.11, p=0.309) | 1.10 (0.99-1.22, p=0.088) | 1.04 (0.88-1.23, p=0.667) |
|  | 3 | 0.96 (0.90-1.03, p=0.301) | 1.01 (0.91-1.13, p=0.806) | 0.98 (0.82-1.16, p=0.783) |
|  | 4 | 0.94 (0.88-1.01, p=0.115) | 0.96 (0.85-1.07, p=0.419) | 0.99 (0.84-1.18, p=0.937) |
|  | 5 | 0.91 (0.84-0.97, p=0.007) | 0.97 (0.87-1.08, p=0.556) | 1.06 (0.90-1.25, p=0.483) |
| Age (years) | <50 | NA | NA | NA |
|  | 50-69 | 1.35 (1.24-1.48, p<0.001) | 1.46 (1.28-1.66, p<0.001) | 1.46 (1.22-1.75, p<0.001) |
|  | 70-79 | 1.36 (1.24-1.49, p<0.001) | 1.27 (1.11-1.45, p=0.001) | 1.08 (0.88-1.31, p=0.475) |
|  | 80+ | 1.12 (1.02-1.23, p=0.014) | 0.86 (0.75-1.00, p=0.044) | 0.62 (0.50-0.76, p<0.001) |
| Respiratory rate** | <20 | NA | NA | NA |
|  | 20-29 | 1.24 (1.17-1.31, p<0.001) | 1.28 (1.18-1.38, p<0.001) | 1.31 (1.16-1.48, p<0.001) |
|  | >30 | 1.68 (1.56-1.81, p<0.001) | 1.88 (1.70-2.07, p<0.001) | 1.97 (1.71-2.26, p<0.001) |
| Oxygen saturation(%)** | >=92 | NA | NA | NA |
|  | <92 | 1.29 (1.21-1.38, p<0.001) | 1.37 (1.24-1.51, p<0.001) | 1.44 (1.21-1.70, p<0.001) |
| Smoking | Never | NA | NA | NA |
|  | Current | 0.98 (0.88-1.09, p=0.658) | 0.91 (0.77-1.07, p=0.265) | 0.90 (0.73-1.12, p=0.338) |
|  | Former | 1.03 (0.97-1.09, p=0.338) | 1.01 (0.92-1.10, p=0.873) | 1.03 (0.89-1.18, p=0.688) |
| Chronic kidney disease | | 1.66 (1.57-1.76, p<0.001) | 1.12 (1.03-1.23, p=0.011) | 0.96 (0.83-1.11, p=0.600) |
| Heart disease | | 0.90 (0.85-0.95, p<0.001) | 0.79 (0.73-0.85, p<0.001) | 0.71 (0.62-0.81, p<0.001) |
| Diabetes | | 1.21 (1.15-1.28, p<0.001) | 1.22 (1.13-1.32, p<0.001) | 1.25 (1.11-1.41, p<0.001) |
| Hypertension | | 1.16 (1.10-1.22, p<0.001) | 1.16 (1.07-1.25, p<0.001) | 1.24 (1.09-1.40, p=0.001) |
| Chronic liver disease | | 0.91 (0.80-1.03, p=0.141) | 0.89 (0.74-1.08, p=0.234) | 0.74 (0.54-1.01, p=0.055) |
| Lung disease (not asthma) | | 0.92 (0.86-0.98, p=0.008) | 0.86 (0.78-0.94, p=0.002) | 0.73 (0.62-0.86, p<0.001) |
| Asthma | | 0.85 (0.80-0.91, p<0.001) | 0.92 (0.84-1.02, p=0.113) | 0.97 (0.84-1.12, p=0.673) |
| Neurological disease | | 0.98 (0.91-1.05, p=0.506) | 0.91 (0.81-1.01, p=0.084) | 0.80 (0.67-0.97, p=0.021) |
| Cancer | | 0.95 (0.88-1.02, p=0.163) | 0.94 (0.84-1.05, p=0.288) | 0.80 (0.66-0.97, p=0.026) |
| Haematological disease | | 0.98 (0.88-1.09, p=0.715) | 0.88 (0.73-1.05, p=0.141) | 0.64 (0.47-0.87, p=0.004) |
| Human immunodeficiency virus | | 1.03 (0.73-1.45, p=0.858) | 1.07 (0.67-1.73, p=0.775) | 1.57 (0.87-2.82, p=0.130) |
| Obesity | | 1.13 (1.06-1.21, p<0.001) | 1.25 (1.14-1.38, p<0.001) | 1.29 (1.12-1.49, p<0.001) |
| Rheumatological disease | | 0.90 (0.84-0.97, p=0.004) | 0.91 (0.81-1.02, p=0.097) | 0.82 (0.69-0.99, p=0.038) |
| Dementia | | 1.15 (1.07-1.23, p<0.001) | 1.01 (0.90-1.13, p=0.853) | 0.80 (0.66-0.96, p=0.020) |
| RAS-blockers | | 1.28 (1.22-1.34, p<0.001) | 1.34 (1.24-1.44, p<0.001) | 1.30 (1.16-1.45, p<0.001) |
| Nonsteroidal anti-inflammatory drugs | | 1.06 (0.94-1.20, p=0.325) | 1.33 (1.13-1.57, p=0.001) | 1.58 (1.26-1.98, p<0.001) |

*Adjusted for age, sex, race, deprivation quintile, chronic kidney disease, heart disease, diabetes, admission oxygen saturations on air and admission respiratory rate

**On admission

Table S5. Interactions between race and other risk factors in kidney replacement therapy logistic regression model

| Interaction | Coefficient |
| --- | --- |
| ethnicityBlack:ckdYes | 0.54 (0.16-0.91, p=0.005) |
| ethnicityEast Asian:ckdYes | 0.52 (-0.53-1.57, p=0.332) |
| ethnicityOther:ckdYes | 0.59 (0.24-0.93, p=0.001) |
| ethnicitySouth Asian:ckdYes | 0.82 (0.51-1.14, p<0.001) |
| ethnicityBlack:diabetesYes | 0.19 (-0.20-0.59, p=0.333) |
| ethnicityEast Asian:diabetesYes | -0.55 (-1.60-0.50, p=0.303) |
| ethnicityOther:diabetesYes | 0.15 (-0.18-0.47, p=0.373) |
| ethnicitySouth Asian:diabetesYes | 0.33 (0.02-0.65, p=0.038) |
| ethnicityBlack:hypertensionYes | 0.21 (-0.18-0.60, p=0.281) |
| ethnicityEast Asian:hypertensionYes | 0.08 (-0.89-1.04, p=0.877) |
| ethnicityOther:hypertensionYes | 0.15 (-0.17-0.46, p=0.358) |
| ethnicitySouth Asian:hypertensionYes | 0.56 (0.24-0.89, p=0.001) |
| ethnicityBlack:sexMale | -0.10 (-0.51-0.31, p=0.631) |
| ethnicityEast Asian:sexMale | -1.10 (-2.03–0.18, p=0.020) |
| ethnicityOther:sexMale | -0.21 (-0.57-0.16, p=0.265) |
| ethnicitySouth Asian:sexMale | -0.46 (-0.79–0.13, p=0.006) |
| ethnicityBlack:RASBYes | 0.08 (-0.30-0.47, p=0.677) |
| ethnicityEast Asian: RASBYes | -0.09 (-1.07-0.89, p=0.864) |
| ethnicityOther: RASBYes | -0.11 (-0.47-0.25, p=0.543) |
| ethnicitySouth Asian: RASBYes | 0.19 (-0.13-0.51, p=0.246) |
| ethnicityBlack:obesityYes | -0.31 (-0.79-0.18, p=0.212) |
| ethnicityEast Asian:obesityYes | -0.40 (-1.74-0.94, p=0.560) |
| ethnicityOther:obesityYes | -0.13 (-0.51-0.25, p=0.513) |
| ethnicitySouth Asian:obesityYes | -0.13 (-0.51-0.24, p=0.483) |
| ethnicityBlack:asthmaYes | -0.28 (-0.88-0.33, p=0.372) |
| ethnicityEast Asian:asthmaYes | -0.29 (-1.76-1.18, p=0.699) |
| ethnicityOther:asthmaYes | -0.17 (-0.60-0.26, p=0.439) |
| ethnicitySouth Asian:asthmaYes | -0.34 (-0.75-0.06, p=0.096) |
| ethnicityBlack:age | 0.01 (0.00-0.02, p=0.010) |
| ethnicityEast Asian:age | 0.02 (-0.00-0.05, p=0.093) |
| ethnicityOther:age | 0.01 (0.01-0.02, p<0.001) |
| ethnicitySouth Asian:age | 0.02 (0.01-0.03, p<0.001) |
| ethnicityBlack:imd_quintile2 | -0.06 (-0.52-0.41, p=0.817) |
| ethnicityEast Asian:imd_quintile2 | 0.48 (-0.93-1.88, p=0.504) |
| ethnicityOther:imd_quintile2 | -0.44 (-0.92-0.03, p=0.069) |
| ethnicitySouth Asian:imd_quintile2 | -0.26 (-0.71-0.18, p=0.248) |
| ethnicityBlack:imd_quintile3 | 0.04 (-0.54-0.62, p=0.891) |
| ethnicityEast Asian:imd_quintile3 | -0.51 (-2.23-1.21, p=0.560) |
| ethnicityOther:imd_quintile3 | -0.26 (-0.76-0.24, p=0.303) |
| ethnicitySouth Asian:imd_quintile3 | 0.13 (-0.35-0.62, p=0.585) |
| ethnicityBlack:imd_quintile4 | -0.23 (-0.84-0.39, p=0.469) |
| ethnicityEast Asian:imd_quintile4 | -0.62 (-2.47-1.23, p=0.509) |
| ethnicityOther:imd_quintile4 | -0.62 (-1.16–0.08, p=0.024) |
| ethnicitySouth Asian:imd_quintile4 | -0.10 (-0.65-0.46, p=0.734) |
| ethnicityBlack:imd_quintile5 | -0.47 (-1.16-0.22, p=0.178) |
| ethnicityEast Asian:imd_quintile5 | 0.09 (-1.41-1.59, p=0.906) |
| ethnicityOther:imd_quintile5 | -0.32 (-0.81-0.17, p=0.200) |
| ethnicitySouth Asian:imd_quintile5 | 0.08 (-0.46-0.62, p=0.768) |
| ethnicityBlack:resp_rate20-29 | -0.07 (-0.47-0.33, p=0.739) |
| ethnicityEast Asian:resp_rate20-29 | 0.45 (-0.93-1.82, p=0.522) |
| ethnicityOther:resp_rate20-29 | 0.03 (-0.34-0.39, p=0.880) |
| ethnicitySouth Asian:resp_rate20-29 | -0.27 (-0.65-0.10, p=0.155) |
| ethnicityBlack:resp_rate>30 | -0.57 (-1.04–0.10, p=0.018) |
| ethnicityEast Asian:resp_rate>30 | 0.24 (-1.08-1.57, p=0.718) |
| ethnicityOther:resp_rate>30 | -0.16 (-0.56-0.25, p=0.448) |
| ethnicitySouth Asian:resp_rate>30 | -0.28 (-0.67-0.11, p=0.161) |
| ethnicityBlack:oxy_sats<92 | -0.36 (-0.80-0.08, p=0.108) |
| ethnicityEast Asian:oxy_sats<92 | -0.32 (-1.62-0.98, p=0.624) |
| ethnicityOther:oxy_sats<92 | -0.19 (-0.61-0.23, p=0.369) |
| ethnicitySouth Asian:oxy_sats<92 | -0.27 (-0.74-0.20, p=0.256) |

Table S6. Interactions between race and other risk factors in biochemical acute kidney injury logistic regression model

| Interaction | Coeffiecient |
| --- | --- |
| ethnicityBlack:ckdYes | 0.38 (0.11-0.66, p=0.007) |
| ethnicityEast Asian:ckdYes | 0.46 (-0.30-1.23, p=0.235) |
| ethnicityOther:ckdYes | 0.23 (0.00-0.46, p=0.049) |
| ethnicitySouth Asian:ckdYes | 0.52 (0.26-0.77, p<0.001) |
| ethnicityBlack:diabetesYes | 0.31 (0.06-0.56, p=0.016) |
| ethnicityEast Asian:diabetesYes | 0.10 (-0.48-0.67, p=0.743) |
| ethnicityOther:diabetesYes | 0.08 (-0.11-0.27, p=0.415) |
| ethnicitySouth Asian:diabetesYes | 0.30 (0.08-0.52, p=0.007) |
| ethnicityBlack:hypertensionYes | 0.15 (-0.09-0.40, p=0.218) |
| ethnicityEast Asian:hypertensionYes | -0.11 (-0.67-0.45, p=0.695) |
| ethnicityOther:hypertensionYes | 0.16 (-0.02-0.35, p=0.085) |
| ethnicitySouth Asian:hypertensionYes | 0.12 (-0.09-0.32, p=0.278) |
| ethnicityBlack:sexMale | -0.00 (-0.24-0.23, p=0.968) |
| ethnicityEast Asian:sexMale | -0.22 (-0.76-0.31, p=0.411) |
| ethnicityOther:sexMale | 0.11 (-0.07-0.29, p=0.249) |
| ethnicitySouth Asian:sexMale | 0.06 (-0.15-0.27, p=0.597) |
| ethnicityBlack: RASBYes | 0.09 (-0.15-0.34, p=0.454) |
| ethnicityEast Asian: RASBYes | -0.39 (-0.93-0.15, p=0.157) |
| ethnicityOther: RASBYes | -0.04 (-0.23-0.15, p=0.669) |
| ethnicitySouth Asian: RASBYes | -0.00 (-0.21-0.21, p=0.984) |
| ethnicityBlack:obesityYes | 0.26 (-0.05-0.57, p=0.101) |
| ethnicityEast Asian:obesityYes | -0.05 (-0.92-0.82, p=0.906) |
| ethnicityOther:obesityYes | 0.22 (-0.03-0.47, p=0.088) |
| ethnicitySouth Asian:obesityYes | 0.02 (-0.26-0.29, p=0.907) |
| ethnicityBlack:dementiaYes | 0.01 (-0.42-0.44, p=0.951) |
| ethnicityEast Asian:dementiaYes | 0.71 (-0.29-1.70, p=0.163) |
| ethnicityOther:dementiaYes | -0.05 (-0.36-0.26, p=0.738) |
| ethnicitySouth Asian:dementiaYes | 0.10 (-0.40-0.59, p=0.701) |
| ethnicityBlack:age | 0.01 (0.00-0.02, p=0.016) |
| ethnicityEast Asian:age | 0.01 (-0.01-0.02, p=0.479) |
| ethnicityOther:age | 0.00 (-0.00-0.01, p=0.140) |
| ethnicitySouth Asian:age | 0.01 (0.00-0.01, p=0.020) |
| ethnicityBlack:imd_quintile2 | -0.23 (-0.53-0.06, p=0.120) |
| ethnicityEast Asian:imd_quintile2 | -0.23 (-1.04-0.58, p=0.576) |
| ethnicityOther:imd_quintile2 | 0.03 (-0.24-0.30, p=0.833) |
| ethnicitySouth Asian:imd_quintile2 | 0.10 (-0.19-0.39, p=0.497) |
| ethnicityBlack:imd_quintile3 | 0.01 (-0.35-0.37, p=0.952) |
| ethnicityEast Asian:imd_quintile3 | -0.26 (-1.08-0.56, p=0.538) |
| ethnicityOther:imd_quintile3 | 0.02 (-0.26-0.30, p=0.898) |
| ethnicitySouth Asian:imd_quintile3 | 0.13 (-0.15-0.41, p=0.372) |
| ethnicityBlack:imd_quintile4 | -0.30 (-0.67-0.08, p=0.125) |
| ethnicityEast Asian:imd_quintile4 | -0.60 (-1.46-0.26, p=0.168) |
| ethnicityOther:imd_quintile4 | -0.16 (-0.45-0.13, p=0.272) |
| ethnicitySouth Asian:imd_quintile4 | 0.24 (-0.10-0.59, p=0.164) |
| ethnicityBlack:imd_quintile5 | -0.24 (-0.64-0.17, p=0.252) |
| ethnicityEast Asian:imd_quintile5 | -0.86 (-1.77-0.05, p=0.065) |
| ethnicityOther:imd_quintile5 | 0.01 (-0.27-0.28, p=0.957) |
| ethnicitySouth Asian:imd_quintile5 | 0.10 (-0.24-0.45, p=0.553) |
| ethnicityBlack:resp_rate20-29 | 0.04 (-0.23-0.30, p=0.786) |
| ethnicityEast Asian:resp_rate20-29 | 0.15 (-0.53-0.82, p=0.664) |
| ethnicityOther:resp_rate20-29 | -0.17 (-0.37-0.03, p=0.096) |
| ethnicitySouth Asian:resp_rate20-29 | -0.30 (-0.53–0.06, p=0.014) |
| ethnicityBlack:resp_rate>30 | -0.21 (-0.53-0.11, p=0.207) |
| ethnicityEast Asian:resp_rate>30 | -0.12 (-0.83-0.59, p=0.744) |
| ethnicityOther:resp_rate>30 | -0.21 (-0.43-0.02, p=0.075) |
| ethnicitySouth Asian:resp_rate>30 | -0.21 (-0.47-0.06, p=0.122) |
| ethnicityBlack:oxy_sats<92 | -0.02 (-0.34-0.30, p=0.899) |
| ethnicityEast Asian:oxy_sats<92 | -0.21 (-0.87-0.44, p=0.515) |
| ethnicityOther:oxy_sats<92 | 0.05 (-0.18-0.27, p=0.671) |
| ethnicitySouth Asian:oxy_sats<92 | 0.06 (-0.21-0.33, p=0.670) |

Table S7. Patient characteristics by race

|  | | **White** | **Black** | **East Asian** | **Other** | **South Asian** |
| --- | --- | --- | --- | --- | --- | --- |
| Age (years) | Median (IQR) | 76 (62 to 84) | 58 (46 to 74) | 60 (46 to 75) | 60 (46 to 76) | 59 (43 to 72) |
| Sex | Female | 28118 (45.0) | 1268 (44.9) | 228 (40.1) | 2295 (42.0) | 1954 (42.0) |
|  | Male | 34259 (54.8) | 1551 (55.0) | 339 (59.7) | 3155 (57.8) | 2692 (57.9) |
| Smoking | Never | 18958 (50.8) | 1424 (79.9) | 302 (79.3) | 2427 (69.4) | 2444 (81.2) |
|  | Current | 3465 (9.3) | 80 (4.5) | 17 (4.5) | 251 (7.2) | 164 (5.5) |
|  | Former | 14920 (40.0) | 278 (15.6) | 62 (16.3) | 820 (23.4) | 400 (13.3) |
| Hypertension | | 31227 (54.2) | 1406 (60.6) | 271 (59.6) | 2265 (50.5) | 2108 (54.7) |
| Diabetes | | 12224 (21.1) | 806 (31.0) | 138 (25.7) | 1259 (25.2) | 1447 (34.4) |
| Chronic kidney disease | | 10589 (17.6) | 427 (15.7) | 52 (9.5) | 655 (12.6) | 649 (14.5) |
| Heart disease | | 21011 (34.8) | 434 (16.0) | 98 (17.8) | 1073 (20.7) | 1060 (23.6) |
| Obesity | | 6613 (12.3) | 383 (15.3) | 45 (9.0) | 626 (13.2) | 569 (14.2) |
| RAS-blockers | | 15782 (27.6) | 651 (29.0) | 147 (33.4) | 1245 (28.3) | 1224 (32.8) |
| Oxygen saturation | Median (IQR) | 96 (93 to 97) | 96 (92 to 98) | 95 (92 to 98) | 96 (93 to 98) | 96 (93 to 98) |
| Respiratory rate | Median (IQR) | 20 (18 to 25) | 22 (18 to 28) | 23 (19 to 30) | 22 (18 to 28) | 22 (19 to 28) |
| Any supplemental oxygen | | 45901 (73.7) | 2123 (75.5) | 433 (76.5) | 4139 (76.1) | 3275 (70.6) |
| Any critical care admission | | 7508 (12.0) | 708 (25.1) | 158 (27.8) | 1217 (22.3) | 1171 (25.2) |
| Any invasive ventilation | | 3688 (5.9) | 449 (15.9) | 111 (19.5) | 772 (14.2) | 549 (11.8) |
| Any non-invasive ventilation | | 8761 (14.1) | 545 (19.3) | 119 (21.0) | 1044 (19.2) | 936 (20.2) |

|  | **February** | **March** | **April** | **May** | **June** | **July** | **August** | **September** | **October** | **November** |
| --- | --- | --- | --- | --- | --- | --- | --- | --- | --- | --- |
| Median days from symptom onset to AKI (IQR) | 3 (2 to 9) | 7 (3 to 12) | 6 (2 to 11) | 4 (1 to 0) | 5 (1 to 9) | 5 (2 to 9) | 6 (4 to 10) | 6 (3 to 10) | 7 (3 to 10) | 7 (4 to 12) |
| Median days from  admission to AKI (IQR) | 2 (0 to 13) | 2 (0 to 5) | 0 (0 to 2) | 0 (0 to 2) | 0 (0 to 2) | 0 (0 to 1) | 0 (0 to 1) | 0 (0 to 1) | 0 (0 to 1) | 0 (0 to 1) |

Table S8. Time to AKI per month in 2020. IQR, interquartile range

Table S9. Medication propensity score matching results

Only patients receiving supplemental oxygen, admitted to hospital after 31/05/2020 and without biochemical acute kidney injury on the day of hospital admission were included. For remdesivir, patients also needed satisfactory kidney and liver function on admission to be included (estimated glomerular filtration rate greater than 30ml/min/1.73m^2^ and alanine aminotransferase less than five times the upper limit of normal).

Exact matching month of admission with nearest neighbour matching for age, sex, race, IMD deprivation quintile, diabetes, heart disease, CKD, RAS-blockers and oxygen saturations on air and respiratory rate on admission. Matching ratio 3:1 where sample size allowed.

1. Dexamethasone Propensity Score Matching

|  | **No biochemical acute kidney injury** | **Biochemical acute kidney injury** | **Odds ratio** |
| --- | --- | --- | --- |
| No dexamethasone (%) | 288 (89.7) | 33 (10.3) | - |
| Dexamethasone (%) | 224 (90.7) | 23 (9.3) | 0.90 (0.51-1.56, p=0.701) |

|  | **No kidney replacement therapy** | **Kidney replacement therapy** | **Odds ratio** |
| --- | --- | --- | --- |
| No dexamethasone (%) | 1775 (99.4) | 11 (0.6) | - |
| Dexamethasone (%) | 1522 (98.6) | 21 (1.4) | 2.23 (1.09-4.80, p=0.032) |

1. Remdesivir Propensity Score Matching

|  | **No biochemical acute kidney injury** | **Biochemical acute kidney injury** | **Odds ratio** |
| --- | --- | --- | --- |
| No remdesivir (%) | 478 (91.0) | 47 (9.0) | - |
| Remdesivir (%) | 376 (92.4) | 31 (7.6) | 0.84 (0.52-1.34, p=0.466) |

|  | **No kidney replacement therapy** | **Kidney replacement therapy** | **Odds ratio** |
| --- | --- | --- | --- |
| No remdesivir (%) | 1635 (99.2) | 14 (0.8) | - |
| Remdesivir (%) | 645 (99.1) | 6 (0.9) | 1.09 (0.38-2.72, p=0.866) |

|  | | **No Dexamethasone**  **N=1473** | **Dexamethasone**  **N=2403** |
| --- | --- | --- | --- |
| Age | Median (IQR) | 70 (54 to 82) | 64 (53 to 76) |
| Sex (%) | Female | 721 (48.9) | 895 (37.2) |
|  | Male | 752 (51.1) | 1500 (62.4) |
| Ethnicity (%) | White | 1066 (82.6) | 1567 (74.4) |
|  | Black | 34 (2.6) | 97 (4.6) |
|  | East Asian | 5 (0.4) | 25 (1.2) |
|  | Other | 105 (8.1) | 171 (8.1) |
|  | South Asian | 80 (6.2) | 246 (11.7) |
| IMD quintile (%) | 1 | 281 (19.4) | 721 (31.0) |
|  | 2 | 295 (20.4) | 504 (21.7) |
|  | 3 | 293 (20.2) | 376 (16.2) |
|  | 4 | 262 (18.1) | 399 (17.2) |
|  | 5 | 317 (21.9) | 325 (14.0) |
| Smoking (%) | Never | 456 (53.5) | 812 (54.1) |
|  | Current | 108 (12.7) | 104 (6.9) |
|  | Former | 288 (33.8) | 585 (39.0) |
| Hypertension (%) | | 660 (50.1) | 1124 (54.4) |
| Diabetes (%) | | 263 (19.3) | 448 (20.4) |
| Chronic kidney disease (%) | | 218 (15.4) | 263 (11.3) |
| Heart disease (%) | | 455 (31.9) | 548 (23.5) |
| Lung disease (not asthma) (%) | | 234 (16.5) | 355 (15.2) |
| Asthma (%) | | 201 (14.1) | 397 (17.0) |
| Chronic liver disease (%) | | 65 (4.6) | 64 (2.8) |
| Neurological disease (%) | | 139 (9.8) | 170 (7.3) |
| Cancer (%) | | 161 (11.4) | 168 (7.3) |
| Haematological disease (%) | | 73 (5.2) | 75 (3.2) |
| Human immunodeficiency virus (%) | | <10 (0.4) | 15 (0.7) |
| Obesity (%) | | 154 (12.1) | 486 (22.8) |
| Rheumatological disease (%) | | 185 (13.1) | 265 (11.4) |
| Dementia (%) | | 165 (11.7) | 95 (4.1) |
| **Treatment before admission** | | | |
| RAS-blockers (%) | | 344 (26.5) | 693 (34.3) |
| NSAIDs (%) | | 67 (5.2) | 93 (4.6) |
| **Treatment during admission** | | | |
| Antibiotics (%) | | 1059 (72.7) | 2214 (93.0) |
| Antifungals (%) | | 55 (3.8) | 179 (7.8) |
| Critical care admission (%) | | 156 (10.6) | 1021 (42.5) |
| Vasoactive drugs (%) | | 35 (2.4) | 213 (9.3) |
| Invasive ventilation (%) | | 61 (4.2) | 336 (14.1) |
| Non-invasive ventilation (%) | | 123 (8.4) | 985 (41.2) |

Table S10. Patient characteristics by administration of dexamethasone status

|  |  | **No Remdesivir**  **N=2719** | **Remdesivir**  **N=1611** |
| --- | --- | --- | --- |
| Age (%) | Median (IQR) | 69 (54 to 80) | 63 (53 to 74) |
| Sex (%) | Female | 1227 (45.1) | 585 (36.3) |
|  | Male | 1484 (54.6) | 1021 (63.4) |
| Ethnicity (%) | White | 1922 (80.7) | 1035 (73.6) |
|  | Black | 75 (3.1) | 62 (4.4) |
|  | East Asian | 15 (0.6) | 18 (1.3) |
|  | Other | 173 (7.3) | 134 (9.5) |
|  | South Asian | 198 (8.3) | 157 (11.2) |
| IMD quintile (%) | 1 | 596 (22.6) | 504 (32.1) |
|  | 2 | 550 (20.8) | 345 (22.0) |
|  | 3 | 516 (19.5) | 248 (15.8) |
|  | 4 | 466 (17.7) | 272 (17.3) |
|  | 5 | 512 (19.4) | 202 (12.9) |
| Smoking (%) | Never | 799 (53.1) | 563 (53.8) |
|  | Current | 178 (11.8) | 66 (6.3) |
|  | Former | 529 (35.1) | 417 (39.9) |
| Hypertension (%) | | 1281 (53.4) | 716 (52.4) |
| Diabetes (%) | | 503 (20.2) | 292 (19.8) |
| Chronic kidney disease (%) | | 420 (16.2) | 120 (7.7) |
| Heart disease (%) | | 794 (30.5) | 331 (21.1) |
| Lung disease (not asthma) (%) | | 414 (16.0) | 260 (16.6) |
| Asthma (%) | | 386 (14.9) | 279 (17.8) |
| Chronic liver disease (%) | | 103 (4.0) | 42 (2.7) |
| Neurological disease (%) | | 256 (9.9) | 99 (6.3) |
| Cancer (%) | | 280 (10.9) | 102 (6.5) |
| Haematological disease (%) | | 123 (4.8) | 42 (2.7) |
| Human immunodeficiency virus (%) | | 12 (0.5) | 11 (0.7) |
| Obesity (%) | | 332 (14.3) | 363 (25.2) |
| Rheumatological disease (%) | | 364 (14.1) | 139 (8.9) |
| Dementia (%) | | 248 (9.6) | 51 (3.2) |
| **Treatment before admission** | | | |
| RAS-blockers (%) | | 693 (29.6) | 453 (33.8) |
| NSAIDs (%) | | 105 (4.5) | 72 (5.4) |
| **Treatment during admission** | | | |
| Antibiotics (%) | | 1992 (79.8) | 1477 (93.4) |
| Antifungals (%) | | 115 (4.8) | 131 (8.5) |
| Critical care admission (%) | | 612 (22.6) | 730 (45.3) |
| Vasoactive drugs (%) | | 107 (4.3) | 147 (9.7) |
| Invasive ventilation (%) | | 189 (7.1) | 243 (15.2) |
| Non-invasive ventilation (%) | | 453 (16.9) | 758 (47.3) |

Table S11. Patient characteristics by administration of remdesivir status
